# Seasonality and fruiting converge on megastigmanes to shape foliar metabolic plasticity in *Miconia albicans* (Swartz) Triana (Melastomataceae)

**DOI:** 10.1007/s11306-026-02436-2

**Published:** 2026-05-06

**Authors:** André Nunes Silva, Djaceli Sampaio de Oliveira Dembogurski, Amanda Galdi Boaretto, Carlos Alexandre Carollo, Flavio Macedo Alves, Denise Brentan Silva

**Affiliations:** 1 Laboratory of Natural Products and Mass Spectrometry (LaPNEM), Faculty of Pharmaceutical Sciences, Food and Nutrition (FACFAN), Federal University of Mato Grosso do Sul, Av. Costa E Silva, S/no, Campo Grande, Mato Grosso do Sul 79070-900 Brazil; 2 Laboratory of Botany, Biosciences Institute (INBIO), Federal University of Mato Grosso do Sul, Campo Grande, Mato Grosso do Sul 79070-900 Brazil

**Keywords:** Megastigmanes, Untargeted metabolomics, *Miconia albicans*, Seasonal differences

## Abstract

**Introduction:**

*Miconia albicans* is a medicinal plant widely used in traditional medicine and represents a promising candidate for phytotherapeutic drug development. Despite its widespread use, the influence of environmental seasonality and phenological state on its foliar metabolome remains poorly understood, particularly in seasonal ecosystems such as the Brazilian savanna (Cerrado Biome).

**Objectives:**

This study aimed to evaluate the influence of seasonality and phenological state on the foliar metabolome of *M. albicans* by untargeted metabolomics and chemometric approaches.

**Methods:**

Leaves of *M. albicans* were collected from the same individuals over an 18-month period, encompassing dry and rainy seasons and contrasting phenological states (fruiting and budding). Samples were analyzed using LC–MS-based untargeted metabolomics, and data were evaluated through multivariate analyses and univariate mixed-effects models.

**Results:**

Twenty-five metabolites were annotated, including flavonoids, megastigmanes, tannins, and triterpenes. Fruiting exerted the strongest effect on global metabolic structure, being associated with marked convergence of foliar metabolomes and coordinated responses of specific chemical classes, such as megastigmanes and tannins. Seasonality mainly affected megastigmanes, which exhibited higher intensities in the dry season. Budding had the weakest effect with responses restricted to the triterpene pathway. Univariate analyses also revealed that all annotated megastigmanes had higher intensities in both fruiting individuals and the dry season.

**Conclusion:**

These results demonstrate that foliar specialized metabolism in *M. albicans* integrates environmental and phenological signals, with megastigmanes participate in a central axis of foliar metabolic plasticity within *M. albicans* under dry and fruiting conditions.

**Supplementary Information:**

The online version contains supplementary material available at 10.1007/s11306-026-02436-2.

## Introduction

*Miconia albicans* (Sw.) Triana (Melastomataceae) is a shrub or small tree that occurs from Mexico to Paraguay and widespread across several Brazilian biomes, including the Amazon, Atlantic Forest, and Caatinga, with predominance in the Cerrado (Goldenberg et al., 2025). The species is popularly known as “canela-de-velho” and used in traditional medicine to treat inflammation, joint pain, infections, and arthritis (Serpeloni et al., 2011). It is widely sold in herbal pharmacies and street markets throughout Brazil (Lima et al., 2018). Several biological properties have been reported for *M. albicans* extracts, including antidiabetic activity (Lima et al., 2018), antioxidant potential (Dembogurski et al., 2023; Lima et al., 2020; Quintans-Júnior et al., 2020), antibacterial and antifungal activities (Celotto et al., 2003).

The chemical composition of *M. albicans* is diverse and includes hydrolysable tannins, triterpenes, steroids, flavonoids, phenolic acids, and their derivatives (Dembogurski et al., 2023; Gimenez et al., 2020; Lima et al., 2018; Quintans-Júnior et al., 2020). More recently, the presence of *O*-glycosylated megastigmanes has also been reported in the aqueous extract from *M. albicans* leaves, together with twenty-two compounds that exhibited inhibition of leukocyte migration, anti-inflammatory, and anti-hyperalgesic properties (Dembogurski et al., 2023).

The accumulation of specialized metabolites can be strongly influenced by biotic and abiotic factors, leading to pronounced changes in metabolic profiles. Consequently, harvest timing represents a critical determinant of chemical composition and, therefore, of the efficacy of medicinal plant extracts (Gobbo-Neto & Lopes, 2007; Li et al., 2020; Yang et al., 2018). Among biotic factors, phenological status has been recognized as a major source of metabolic variation. In the medicinal plant *Angelica sinensis* (Oliv.) (Apiaceae), for example, early flowering induces root lignification and reduces the accumulation of bioactive metabolites, such as ferulic acid and ligustilide, thereby compromising medicinal quality (Li et al., 2022).

Abiotic factors, particularly climatic seasonality, also play a central role in modulating plant metabolism (Prinsloo & Nogemane, 2018). *M. albicans* occurs widely in the Brazilian Savanna (Cerrado), a phytogeographic domain characterized by strong climatic seasonality, with well-defined dry and rainy seasons and pronounced environmental fluctuations, including marked variation in water availability, high daily thermal amplitude, intense solar and UV radiation, seasonal drought-associated oxidative stress, and recurrent fire regimes. Together, these factors impose strong selective pressures on plant metabolism, particularly on redox-regulated pathways and specialized metabolite production (Eiten, 1972). Studies conducted in this biome have shown that seasonal variation can modulate the metabolism of different plant species. For example, in *Duguetia furfuracea* (A.St.-Hil.) Saff. (Annonaceae) the alkaloid levels vary throughout the year, whereas flavonoid accumulation is higher during the rainy season (Macedo et al., 2021). Similarly, the metabolism of *Lychnophora ericoides* Mart. (Asteraceae) is also modulated by seasonality, exhibiting higher concentrations of chlorogenic acids during the dry season (Gobbo-Neto et al., 2017).

The leaf chemical composition of *M. albicans* was investigated for seasonal and geographic variation across three Cerrado sites in the state of São Paulo, with five individuals sampled per site during one month of both the dry and the rainy seasons (Gimenez et al., 2020). The study was based on a targeted analytical design, quantifying a restricted set of previously described compounds by HPLC–DAD. Quantitative analyses of leaf extracts revealed higher levels of the flavonols rutin and quercetin during the rainy season, whereas the concentrations of the triterpenes oleanolic and ursolic acids decreased (Gimenez et al., 2020). These observations suggest adaptive responses in *M. albicans,* although the global metabolome was not assessed, and sampling was limited to a single month per season.

Despite growing evidence that seasonal and phenological factors influence plant chemical composition, the knowledge regarding the variability of specific metabolite classes in *M. albicans* remains limited. Megastigmanes are structurally diverse C13-norisoprenoids formed from carotenoids via non-enzymatic or enzymatic reactions catalyzed by dioxygenases, or via glycosylation-associated degradation pathways (Samra et al., 2024). Although several megastigmanes have been reported to exhibit relevant biological activities, including antibacterial, hepatoprotective, antiviral, neuroprotective, and anti-inflammatory properties (Bao et al., 2017; El-Sayed et al., 2025; Thao et al., 2015), this class of metabolites remains poorly explored in plant metabolomic and ecological studies. In particular, information on their occurrence, regulation, and ecological significance in the genus *Miconia* is extremely limited, and the factors controlling their accumulation in *M. albicans* have not yet been systematically investigated (Dembogurski et al., 2023).

Considering that seasonality and phenological status can modulate plant specialized metabolism, these factors constitute critical variables for the quality control of medicinal species such as *M. albicans*. In this context, metabolomics provides an appropriate framework for integrated monitoring of chemical composition by enabling comprehensive characterization of metabolites across different biological levels. Advances in analytical techniques (GC–MS, LC–MS, NMR), combined with statistical and chemometric methods, allow for the accurate detection of metabolic variation, robust comparison among samples, and the association of metabolic profiles with environmental and seasonal factors (Allwood et al., 2021; De Vos et al., 2007).

Accordingly, this study aims to investigate the effects of seasonality (dry and rainy seasons) together with phenological status (fruiting and budding) on the specialized metabolism of *M. albicans* in the Brazilian Savanna using an untargeted metabolomics approach integrated with chemometric tools.

## Materials and methods

### Study area

Leaves of *M. albicans* were collected in a Private Natural Heritage Reserve (Latitude 20°30′29.99″ S, Longitude 54°36′58.43″ W) located at the Federal University of Mato Grosso do Sul (UFMS), in Campo Grande, Mato Grosso do Sul, Brazil (Fig. S1). This reserve comprises an urban fragment of approximately 32 hectares of Brazilian savanna (Cerrado). The climate is classified as Aw according to the Köppen system, characterized by a dry winter with low precipitation, followed by a rainy summer during which most of the annual rainfall occurs (Lima et al., 2023). The local phytophysiognomy includes forested vegetation known as “cerradão”, characterized by tall trees, as well as open areas dominated by shrubs, herbs, and grasses. *M. albicans* was found both at forest edges and within the forest interior, particularly in canopy gaps, where conditions favor the establishment and growth of pioneer species such as *M. albicans*.

### Plant material

We collected mature leaves from four *M. albicans* individuals over ten months spanning August 2018 to February 2020, totaling 40 sampling events. Sampling was conducted in the afternoon from plants at similar developmental stages, with material collected from different positions within each individual. Leaf material was subsequently stabilized by drying in a forced-air oven at 50 °C for 48 h, powdered using a knife mill, and stored under vacuum in sealed bags at − 20 °C until chemical analyses to minimize oxidative processes and metabolite degradation. The phenological stages of the individuals were monitored, and the presence of flowers, buds, fruits, and newly emerged leaves was recorded (Table S1). A voucher specimen was deposited at the herbarium of Federal University of Mato Grosso do Sul, Campo Grande, Mato Grosso do Sul, Brazil (GCMS); acronym registered in the Index Herbariorum under accession number CGMS 79407, in compliance with authorization from SisGen (National System for the Management of Genetic Heritage and Associated Traditional Knowledge), registration protocol number ACD1712.

### Climate information

Climatic data of the months of collection (August 2018 to February 2020) were obtained from the available online database of Mato Grosso do Sul Weather and Climate Monitoring Center (CEMTEC, 2019). The acquired data were the instantaneous temperature (IT), wind gust (WG), minimum temperature (MAINT), rainfall (RA), days without rain (DWR), maximum temperature (MAXT), maximum humidity (MAXH), minimum humidity (MINH), and instantaneous humidity (IH) for each month of sampling (Table S2).

### Sample preparation

An aliquot of the dried powdered leaf material was further ground using a mixer mill (Retsch® MM 400, Haan, Germany) to standardize particle size. These samples were then extracted with a mixture of methanol and ultrapure water 7:3 (v/v) at a concentration of 10 mg/mL using an ultrasonic bath (Elma®, Elmasonic E 30 H, power 240 W, Singen, Germany) for 10 min. The extracts were subsequently centrifuged at 10,000 rpm for 3 min (Kasvi®, model K14-1215, power 85 W, Zhejiang, China), and the supernatant was carefully collected and filtered through PTFE syringe filter (Millex 0.22 µm × 13 mm, Millipore®, Burlington, MA, USA) before injection into the chromatographic system. The quality control (QC) was prepared by mixing 25 µL of each sample extract, which was then homogenized and injected after every five analytical samples in the metabolomic experiment.

### Liquid chromatography coupled to diode array detector and mass spectrometer (LC-DAD-MS) analysis

The samples, including blanks and QC, were analyzed by an UFLC LC-20AD Shimadzu Prominence® coupled to a diode array detector and a high-resolution mass spectrometer with electrospray ionization source (MicrOTOF-Q III – Bruker Daltonics®, Billerica, MA, USA). Sample injections were performed in a random order. The chromatographic analyses were achieved on a Kinetex® C18 column (00F-4462-AN, 2.6 µm, 100 Å, 150 × 2.1 mm, Phenomenex®, Torrance, CA, USA), which was maintained at 50 °C in the analyses, and a flow rate of 0.3 mL/min was applied. Acetonitrile (solvent B) and ultrapure water (solvent A), each containing 0.1% formic acid (v/v), were used as mobile phase and the following elution gradient program was used: 0–2 min (3% of B), 2–25 min (3 to 25% of B), 25–40 min (25 to 80% of B), and 40–43 min (80% of B). The MS analyses were performed in negative ion mode for the data processing, which was previously determined from the QC analyses in positive and negative ion mode. N_2_ was used as nebulizer (pressure 4 Bar), dry (flow rate 9 L/min and temperature 200* m/z*. Sodium formate solution (10 mM NaOH solution in isopropanol: water (1:1), containing 0.2% formic acid) was directly injected at the end of each chromatographic run for internal calibration.

The QC samples were additionally analyzed in data-dependent acquisition (DDA) mode in both positive and negative ion modes. The most intense precursor ions (top 5 per cycle) were automatically selected based on MS^1^ signal intensity and fragmentated by CID. MS^1^ and MS^2^ acquisition was performed over the *m/z* range of 50–1500. The MS/MS were acquired only from QC samples.

### Metabolomics data processing

Initially, the raw LC–MS data were analyzed using DataAnalysis 4.2 software (Bruker Daltonics®). Subsequently, the files were processed in MetAlign 3.0 (Lommen, 2009; Lommen & Kools, 2012), which included baseline correction, noise reduction, peak detection, and alignment, as well as mass assignment to the detected signals. This preprocessing step resulted in 1,003 aligned mass signals (*m/z*–retention time pairs), which were subsequently grouped using MSClust (Tikunov et al., 2012). This algorithm clusters correlated ions originating from the same metabolite into representative pseudo-compounds. Thus, the MSClust analysis resulted in a dataset with 122 features. Finally, to further refine the dataset, signals detected in blank samples were removed, as well as duplicate peaks and features corresponding to adducts that remained in the dataset. In addition, low-intensity ions (threshold set at 3,000) were filtered out, yielding a final dataset comprising 25 representative features used for subsequent statistical analyses. All features obtained after data processing were included in the statistical analyses, and metabolite annotation was subsequently performed.

### Statistical analysis

Statistical analyses were performed in the R environment (version 4.1.3) (RCoreTeam, 2022). The metabolic data matrix consisted of relative intensities of individual metabolites and chemical classes obtained after LC–MS data preprocessing. Before statistical analyses, the data were log-transformed (log_10_) to reduce skewness and the influence of extreme values.

The effects of seasonality, fruiting, and budding were tested additively using permutational multivariate analysis of variance (PERMANOVA) based on Bray–Curtis dissimilarity matrices, implemented with the adonis2 function from the vegan package. To account for the non-independence of samples derived from the same individuals, permutations were stratified by individual. Homogeneity of multivariate dispersion among groups was assessed using the betadisper test, followed by a permutation test. Multivariate variation associated with seasonality, fruiting, and budding was visualized using Principal Coordinates Analysis (PCoA), also based on Bray–Curtis distances.

To evaluate the effects of seasonality, fruiting, and budding on individual metabolites, linear mixed-effects models were fitted using the lme4 package. Log_10_-transformed metabolic intensities were used as response variables, while Season, Fruit, and Bud were included as fixed effects, and individual identity was treated as a random effect. The overall significance of fixed effects was assessed using likelihood ratio tests (LRT), comparing full models with null models containing only the random effect, as well as through residual diagnostics based on the DHARMa package, including tests of uniformity, dispersion, and the presence of outliers.

Estimates of fixed effects were obtained using the lmerTest package, with degrees of freedom estimated by the Satterthwaite method. Ninety-five percent confidence intervals were calculated based on the critical t value. For analyses involving multiple metabolites, p values were adjusted using the Benjamini–Hochberg method to control the false discovery rate (FDR). The effects of seasonality, fruiting, and budding on individual metabolites were visualized using forest plots.

Additionally, to explore the multivariate structure of the data and identify metabolites associated with group discrimination, a partial least squares discriminant analysis (PLS-DA) was performed using metabolite intensities previously adjusted for individual-level effects. PLS-DA models were fitted in the R environment using the mixOmics package. Model complexity was optimized using repeated M-fold cross-validation (5 folds, 50 repetitions), selecting the number of latent components that minimized the balanced error rate (BER). Model performance was further assessed based on mean classification accuracy and area under the receiver operating characteristic curve (AUC), both estimated within the cross-validation framework, ensuring a robust evaluation independent of data partitioning.

To assess the statistical significance of the model and control for overfitting, a permutation test was conducted. In this procedure, class labels were randomly shuffled (n = 999 permutations) while preserving the structure of the data matrix. For each permutation, a new PLS-DA model was fitted using the same number of latent components defined for the original model, and its performance was re-estimated using M-fold cross-validation (5 folds, 10 repetitions). Thus, cross-validation was reapplied to each permuted dataset, rather than being restricted to the original model, ensuring that the null distributions of BER and accuracy incorporated the full model fitting and validation procedure. This approach provides a more rigorous assessment by accounting simultaneously for class label randomization and the variability associated with model training and resampling.

The contribution of individual metabolites to group discrimination was assessed using variable importance in projection (VIP) scores, with metabolites showing VIP > 1.0 considered relevant. To strengthen the robustness of the interpretation, multivariate results were integrated with estimates derived from previously fitted linear mixed-effects models, allowing the identification of metabolites with consistent effects across both multivariate and univariate analyses. For univariate inference, p-values were adjusted using the Benjamini–Hochberg procedure, adopting a false discovery rate (FDR) threshold of p < 0.05.

### Annotation of the metabolites

Metabolite annotation was performed by comparing the acquired spectral data (UV, MS, and MS/MS) with published data (Dembogurski et al., 2023) and spectra deposited in GNPS and SIRIUS. The levels of metabolite annotation were assigned and presented in Table 1 according to the Metabolomics Standards Initiative guidelines (Sumner et al., 2007). Accurate MS data were used to determine the molecular formula, considering errors and mSigma up to 8 ppm and 30, respectively. The spectral data of the compounds were compared with published data, and searches for the proposed compounds were performed using the SciFinder (Chemical Abstracts) and LOTUS databases. In addition, the injection of authentic standards (citric acid, ellagic acid, epicatechin, and rutin) was performed to confirm the annotation.

## Results

### Metabolite annotation from *M. albicans*

From the leaf samples, twenty-five metabolites were annotated (Table 1 and Fig. S2), including *O*-glycosylated flavonols (P12, P13, P15, and P17), triterpenes (P20, P21, P22, and P23), hydrolysable tannins (P3, P4, P5, P6, and P7), megastigmanes (P9, P10, P14, and P16), ellagic acid (P11) and its derivatives (P18 and P19). Megastigmanes P14 and P16 were detected for the first time in the genus *Miconia*.

**Table 1:** Compounds annotated from *Miconia albicans* leaves by LC-DAD-MS

Peak	RT (min)	MF	Compound	Class	Annotation Level^**^	UV (max)	Negative (*m/z*)		Positive (*m/z*)	
							MS[M-H]^−^ (error, mSigma)	MS/MS	MS[M + H]^+^	MS/MS
P1	1.2	C_12_H_22_O_11_	di-*O*-hexoside	Sugar	2	–	341.1089 (2.4, 2.0)	–	–	–
P2	1.5	C_6_H_8_O_7_	Citric acid*	Organic acid	1	–	191.0204 (3.5, 7.8)	–	–	–
P3	3.6	C_41_H_26_O_26_	NHTP-HHDP-hexoside (vescalagin or castalagin isomer)	Tannin	2	282	933.0633 (0.7, 9.2)	467, 421, 301, 275, 257, 231	935.0720	469, 439, 307, 277
P4	5.2	C_41_H_26_O_26_	NHTP-HHDP-hexoside (vescalagin or castalagin isomer)	Tannin	2	280	933.0663 (2.5, 9.2)	467, 421, 301, 275, 257, 231	935.0731	615, 495, 469, 439, 307, 277
P5	7.1	C_42_H_28_O_26_	*O*-methyl NHTP-HHDP-hexoside (*O*-methyl vescalagin or castalagin isomer)	Tannin	2	280	947.0780 (1.7, 6.3)	493, 467, 421, 301, 275, 203	949.0886	–
P6	7.6	C_41_H_24_O_25_	Hydrolysable tannin	Tannin	3	280	915.0516 (1.9, 8.3)	457, 169	–	–
P7	10.3	C_56_H_38_O_31_	C-procyanidin NHTP-HHDP-hexoside^#^	Tannin	2	280	602.0624^–2^ (0.2, 4.3)	493, 467, 301, 289, 275, 249	–	–
P8	13.2	C_15_H_14_O_6_	Epicatechin*	Flavonoid	1	280	289.0715 (0.9, 7.8)	163	291.0861	–
P9	14.6	C_19_H_30_O_8_	Dihydroxy-megastigmadienone *O*-hexoside (roseoside)	Apocarotenoid (megastigmane)	2	–	385.1858 (2.5, 17.5)	205, 153	387.2031	207, 189, 161
P10	18.2	C_19_H_34_O_8_	Trihydroxy megastigmanene *O*-hexoside	Apocarotenoid (megastigmane)	2	–	389.2174 (1.8, 2.5)	189	391.2340	229, 211, 193, 175
P11	18.5	C_14_H_6_O_8_	Ellagic acid*^#^	Phenolic acid	1	250, 365	300.9990 (0, 0.1)	283, 245, 229, 217, 201, 185, 173, 161	303.0172	–
P12	19.3	C_21_H_20_O_12_	Quercetin *O*-hexoside^#S^	Flavonoid	2	270, 356	463.0882 (0.1, 2.0)	300, 271, 255, 243, 179, 151	465.1003	303
P13	19.4	C_27_H_30_O_16_	Rutin*^#S^	Flavonoid	1	270, 352	609.1454 (1.2, 7.3)	463, 301, 151	677.1591	465, 303
P14	19.5	C_26_H_34_O_12_	Mallophenol B^S^	Apocarotenoid (megastigmane)	2	280	537.1970 (1.4, 6.0)	385, 313, 271, 223, 211, 169	539.2107	–
P15	19.8	C_21_H_20_O_12_	Quercetin *O*-hexoside^#^	Flavonoid	2	270, 356	463.0885 (0.5, 0.9)	300, 271, 255, 151	465.1003	303
P16	21.2	C_26_H_38_O_12_	Clypearoside A	Apocarotenoid (megastigmane)	2	280	541.2281 (1.7, 8.0)	371, 363, 331, 313, 271, 253, 227, 169	543.2459	–
P17	22.1	C_21_H_20_O_11_	Quercetin *O*-deoxyhexoside^#S^	Flavonoid	2	262, 350	447.0925 (1.8, 2.6)	300, 271, 255, 163	449.1057	303, 257, 229
P18	22.9	C_21_H_18_O_12_	*O*-deoxyhexosyl *O*-methyl ellagic acid^S^	Phenolic acid	2	275, 365	461.0727 (0.4. 6.4)	315, 300	463.0910	–
P19	25.4	C_17_H_12_O_8_	Tri-*O*-methyl ellagic acid^S^	Phenolic acids	2	272, 365	343.0461 (0.5, 1.4)	311, 298	345.0585	330, 315, 300, 285
P20	30.7	C_36_H_58_O_11_	*O*-hexosyl triterpene^#^	Triterpene	3	–	711.3960^#^ (0.2, 9.9)	503	689.3878^Na^	505, 487, 469, 451, 439, 405, 261, 215, 187
P21	30.8	C_36_H_58_O_11_	*O*-hexosyl triterpene^#^	Triterpene	3	–	711.3959^#^ (0.3, 11.8)	503	689.3878^Na^	505, 487, 469, 451, 439, 405, 261, 215, 187
P22	31.7	C_36_H_58_O_10_	*O*-hexosyl triterpene^S^	Triterpene	3	–	695.4003 (1.7, 7.3)	487	–	–
P23	35.5	C_30_H_48_O_5_	Triterpene (arjunolic acid)	Triterpene	3	–	487.3431 (0.3, 6.0)	409	–	–
P24	39.6	C_28_H_44_O_11_	Unknown	–	4	–	555.2846 (6.3, 13.4)	299, 255, 225, 206, 164	–	–
P25	39.6	C_25_H_46_O_10_	Fatty acid derivative	Fatty acid	3	–	505.3025 (1.3, 3.1)	255, 249	–	–

RT (min): retention time (minute); MF: molecular formula; UV (max): maximum absorption ultraviolet; *: confirmed by injection of authentic standard; ^**^: Identification level for metabolites according to Metabolomics Standards Initiative (MSI): 1 (confirmed with standard); 2 (putatively annotated); 3 (putative class); 4 (unknown).; ^#^: confirmed by MS/MS spectral matching against GNPS library databases (cosine score > 0.7); ^S^: Annotation confirmed by computational fragmentation analysis implemented in SIRIUS; HHDP: hexahydroxydiphenoyl; NHTP: nonahydroxytriphenoyl; ^−2^: [M-2H]^−2^; ^#^: [M-H + HCOOH]^−^

The compounds P1 and P2 revealed intense deprotonated ions at *m/z* 341.1061 (C_12_H_22_O_11_) and 191.0229 (C_6_H_8_O_7_), respectively. The metabolites P1 and P2 were annotated as di-*O*-hexoside and citric acid, besides the compound P2 was also confirmed by an authentic standard.

The metabolite P8 revealed an absorption band at 280 nm in the UV spectrum, which is consistent with flavan-3-ol chromophores. The compound P8 showed a deprotonated ion at *m/z* 289.0709 (C_15_H_13_O_6_^−^) and a fragmentation pathway compatible with epicatechin (Dembogurski et al., 2023). This metabolite was confirmed by the injection of an authentic standard of epicatechin (P8). The metabolites P3**-**P7 showed similar UV spectra with absorption bands at the wavelength ≈ 280 nm. They exhibited molecular formulas corresponding to C_41_H_26_O_26_ (*m/z* 933.0615 [M-H]^−^), C_42_H_28_O_26_ (*m/z* 947.0811 [M-H]^−^), C_41_H_24_O_25_ (*m/z* 915.0478 [M-H]^−^), and C_56_H_38_O_31_ (*m/z* 602.0252 [M-H]^−2^). These metabolites exhibited fragment ions at *m/z* 301, corresponding to hexahydroxydiphenyl (HHDP). These units can undergo rearrangement to form ellagic acid during mass spectrometric analysis; however, the UV spectral data confirmed the presence of HHDP units (Cerulli et al., 2020; Rasines-Perea et al., 2019). In addition, compound P7 showed a product ion at *m/z* 289, suggesting the presence of a procyanidin unit. Thus, these metabolites were annotated as NHTP-HHDP-xoside (vescalagin or castalagin isomer) (P3), NHTP-HHDP-hexoside (vescalagin or castalagin isomer) (P4), *O*-methyl NHTP-HHDP-hexoside (*O*-methyl vescalagin or castalagin isomer) (P5), and *C*-procyanidin NHTP-HHDP-hexoside (P7). The compound P6 was annotated as a hydrolysable tannin.

The peaks P9 and P10 showed no UV absorption, and their protonated ions at *m/z* 387.2031 and 391.2340 suggested the molecular formulas C_19_H_30_O_8_ and C_19_H_34_O_8._ They exhibited losses of an *O*-hexosyl group (162 *u*) yielding the fragment ions *m/z* 207 [M + H-162-H_2_O]^+^ and 229 [M + H-162]^+^, respectively. These aglycone fragment ions are consistent with C13 megastigmanes (Jo et al., 2020), which were annotated as dihydroxy-megastigmadienone *O*-hexoside (roseoside) and trihydroxy-megastigmanene *O*-hexoside (Chang et al., 2020; Spínola et al., 2015). In addition, compounds P14 and P16 showed absorption bands at λ_max_ ≈ 280 nm in the UV spectra, while their deprotonated ions at *m/z* 537.1966 and 541.2277 were compatible with the molecular formulas C_26_H_34_O_12_ and C_26_H_38_O_12_. These compounds showed losses of galloyl units yielding the ions at *m/z* 385 [M-H-galloyl]^−^ and 371 [M-H-galloyl-H_2_O]^−^ for P14 and P16, respectively. Additionally, the product ions *m/z* 313 [*O*-hexosyl gallic acid-H]^−^ also indicated the galloyl substituent linked to hexosyl, as well as the ions *m/z* 169 [gallic acid-H]^−^ for both compounds. The fragment ions of aglycones *m/z* 223 [C_13_H_20_O_3_-H]^−^ and 227 [C_13_H_24_O_3_-H]^−^ confirmed the units of C13 megastigmanes (Dembogurski et al., 2023; Li et al., 2015; Wei et al., 2004).

The chromatographic peaks P12**-**P13, P15, and P17 showed two absorption bands at the wavelengths λ_max_ ≈ 270 and 350 nm in their UV spectra, which are similar for flavonol chromophores. The losses of 162 and 146 *u* indicated the *O*-hexosyl and *O*-deoxyhexosyl substituents, respectively (Silva et al., 2013). The fragment ions of aglycones were observed at *m/z* 300 [quercetin-H]^−•^ and their fragmentation pathway was compatible with quercetin (Younis et al., 2020). Thus, they were annotated as *O*-hexosyl quercetin (P12 and P15), rutin (P13), and *O*-deoxyhexosyl quercetin (P17). The flavonol rutin was confirmed by injection of an authentic standard.

The UV spectra of metabolites P18 and P19 exhibited two absorption bands at λ_max_ ≈ 250 nm and 370 nm, similar to that observed for the ellagic acid chromophore (Reichert et al., 2018). From the protonated ion *m/z* 345.0585 of compound P19, the fragment ions at *m/z* 330, 315, and 300 were observed, which are yielded from subsequent losses of radical methyl (15 *u*), confirming the presence of three methoxyl groups in the structure of ellagic acid (Moilanen et al., 2013; Reichert et al., 2018). In addition, the deprotonated ion *m/z* 461.0727 of compound P18 exhibited a loss of deoxyhexosyl (146 *u*) and subsequent loss of a radical methyl (15 *u*) to yield the fragment ion at *m/z* 300. Thus, these compounds were annotated as *O*-deoxyhexosyl *O*-methyl ellagic acid (P18) and tri-*O*-methyl ellagic acid (P19).

The compounds P20**-**P23 revealed no UV absorption. P20 and P21 showed the same molecular formula (C_36_H_58_O_11_) from their deprotonated ions at *m/z* 711.3984 and 711.3980 [M-H + HCOOH]^−^. The fragment ions at *m/z* 503.3396 (C_30_H_47_O_6_^−^) are yielded from losses of 208 *u* (162 *u* + HCOOH), suggesting a hexosyl substituent and indicating the aglycone triterpenic. Additionally, consecutive losses of water molecules were also observed in positive ion mode, such as the fragment ions at *m/z* 487 [M + H-162-H_2_O]^+^, 469 [M + H-162-2xH_2_O]^+^, and 451 [M + H-162-3xH_2_O]^+^. Similar spectral data were observed for triterpene P22, but this compound showed one less hydroxyl group compared to P20 and P21. Therefore, the compounds P20**-**P22 were annotated as *O*-hexosyl triterpenes (Gao et al., 2011). The compound P23 presented a deprotonated ion at *m/z* 487.3431 [M-H]^−^ and molecular formula C_30_H_48_O_5_, which exhibited the fragment ion at *m/z* 409 and was assigned arjunolic acid (Correa et al., 2021).

### Metabolomics analyses from *M. albicans*

PERMANOVA revealed significant effects of seasonality, fruiting, and budding on the foliar metabolic composition of *M. albicans* (Season: Pseudo-F_1_,_36_ = 5.06, R^2^ = 0.0970, p = 0.0013; Fruit: Pseudo-F_1_,_36_ = 8.45, R^2^ = 0.1619, p = 0.0002; Bud: Pseudo-F_1_,_36_ = 2.67, R^2^ = 0.0511, p = 0.0290). Fruiting exhibited the largest effect size, explaining 16.2% of the multivariate variation, followed by seasonality (9.7%) and budding (5.1%). Collectively, these factors accounted for 31.0% of the total metabolic variation. A Principal Coordinates Analysis (PCoA) including all samples was also constructed to visualize overall multivariate patterns and is presented in Fig. S3.

Model adequacy was supported by predominantly non-significant results in simulation-based residual diagnostics (DHARMa), including tests of uniformity, dispersion, and outliers. In addition, likelihood ratio tests comparing full models to null models indicated that inclusion of phenological factors resulted in significant improvements in model fit for several metabolites. Estimates of marginal (R^2^m) and conditional (R^2^c) coefficients of determination indicated consistent contributions of seasonality, fruiting, and budding to the observed variance. Subsequent interpretation focused on the isolated evaluation of each phenological factor within statistically adequate models (Table S3).

### Seasonality-associated variation in the foliar metabolome

Once PERMANOVA indicated a significant association between seasonality and the multivariate metabolic structure of leaves (Season: Pseudo-F_1_,_36_ = 5.06, R^2^ = 0.0970), we evaluated the homogeneity of multivariate dispersions. PERMDISP revealed significant heterogeneity of dispersion (Pseudo-F_1_,_38_ = 8.832, p = 0.004), indicating that samples from the rainy season exhibit greater multivariate dispersion around their centroid compared with those from the dry season. This difference is corroborated by the higher mean distance to the multivariate median observed in the rainy season (0.02088) relative to the dry season (0.01519). In this context, the seasonal significance detected by PERMANOVA reflects global multivariate differences between seasons that manifest primarily as increased metabolic heterogeneity, rather than as a uniform shift in the average metabolic profile between groups.

**Fig. 1:**
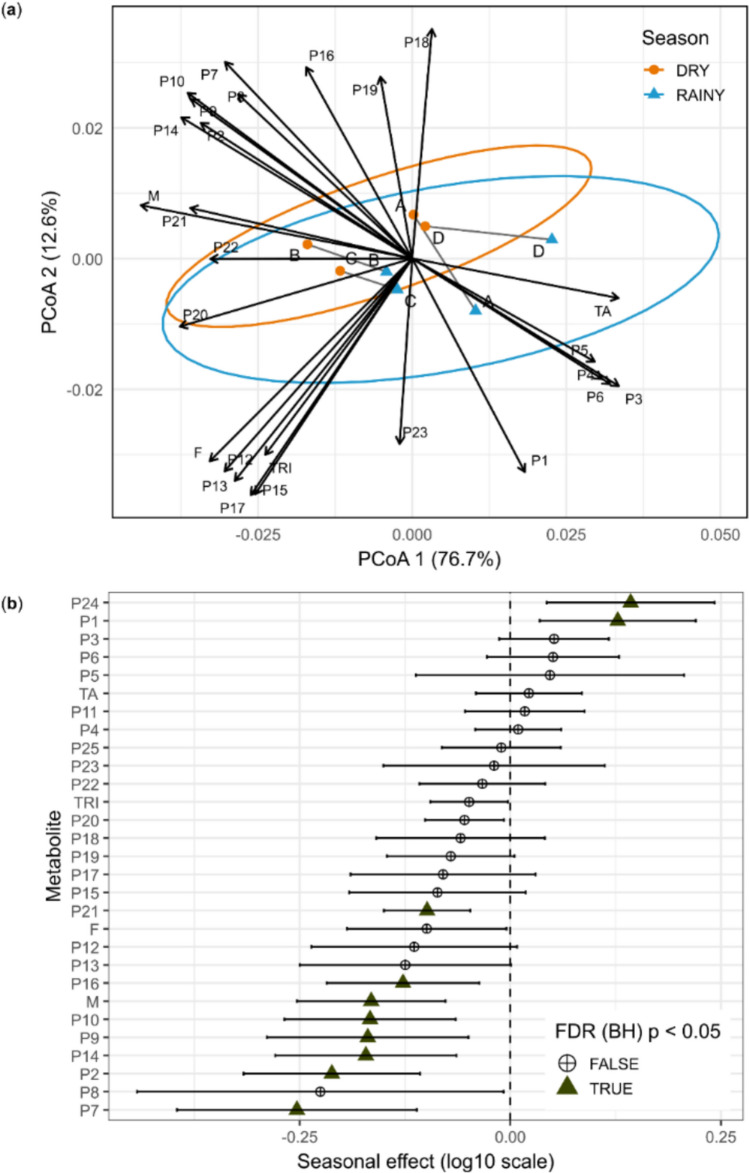
**a** Principal coordinates analysis (PCoA) based on Bray–Curtis distances calculated from metabolite intensities (log_10_ scale), showing the separation between samples collected during the dry (DRY) and rainy (RAINY) seasons. Points represent individual plants (A, B, C, or D), connected by grey lines to indicate paired sampling over time. **b** Forest plot of the fixed effects of seasonality estimated using linear mixed-effects models (log_10_ scale), with individual included as a random effect. Points represent effect estimates, and horizontal bars indicate 95% confidence intervals based on the t distribution. Positive values indicate higher mean intensities during the rainy season, whereas negative values indicate higher mean intensities during the dry season. Metabolites showing significant effects after multiple-testing correction using the Benjamini–Hochberg method (FDR p < 0.05) are indicated by triangles; circles denote non-significant effects. The annotations of the compounds (P1-P25) are described in Table 1. *F* flavonoids, *M* megastigmanes, *TA* hydrolysable tannins, *TRI* triterpenes

To explore the structure of multivariate patterns, we performed a principal coordinates analysis (PCoA) based on Bray–Curtis distances, using the median metabolite intensities for each individual in each season (Fig. 1a). The first two ordination axes jointly explained 89.3% of the variation associated with dissimilarities among samples (PCoA1 = 76.7%; PCoA2 = 12.6%). The resulting ordination revealed greater dispersion of points associated with the rainy season in multivariate space, corroborating the increased metabolic dispersion indicated by PERMDISP.

Although the multivariate metabolic profile of *M. albicans* leaves is heterogeneous between seasons, inference at the level of individual metabolites reveals clear seasonal responses. Previously validated mixed-effects models were used to extract, for each peak, the estimated fixed effect of seasonality. Seasonal effect estimates (rainy–dry contrast) derived from the models were summarized in a forest plot as point estimates with Wald 95% confidence intervals (Fig. 1b). After p-value adjustment using the Benjamini–Hochberg procedure (FDR < 0.05), metabolites with significant effects were identified, and their boxplots are shown in Fig. S4.

Megastigmanes (P9, P10, P14, and P16), *O*-hexosyl triterpene (P21), tannin *C*-procyanidin NHTP–HHDP–hexoside (P7), and citric acid (P2) exhibited negative coefficients in the seasonal contrast, indicating higher mean intensities during the dry season. Among the detected chemical classes, only megastigmanes (M) showed a consistent seasonal response, with increased intensities observed for all metabolites of this class during the dry period. In contrast, the sugar P1 (di-*O*-hexoside) and the unidentified peak (P24) displayed positive coefficients, indicating higher intensities during the rainy season.

**Fig. 2:**
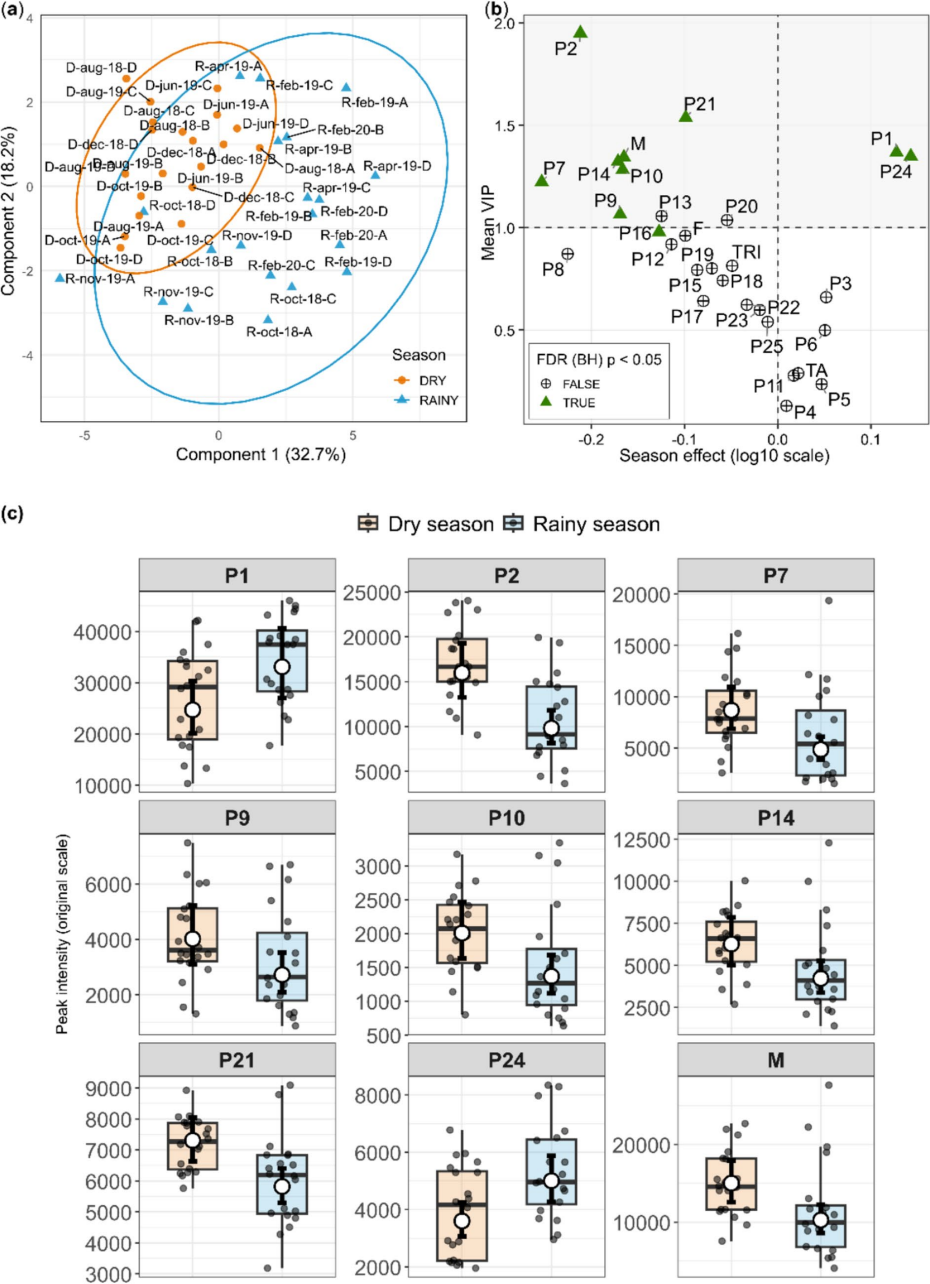
**a** PLS-DA score plot based on metabolite intensities adjusted for individual-level effects, showing a partial separation between dry (orange) and rainy (blue) season samples. Ellipses represent 95% confidence regions. The first two components explain 32.7% and 18.2% of the variance, respectively. **b** Integration of multivariate and univariate results. Mean VIP scores (y-axis) are plotted against the estimated seasonal effect (rainy–dry contrast, log10 scale; x-axis) derived from linear mixed-effects models. Metabolites above the horizontal dashed line (VIP > 1) that remained significant after FDR correction (Benjamini–Hochberg, p < 0.05) were considered relevant for multivariate discrimination. Metabolites significant after FDR correction are highlighted with green triangles. **c** Boxplots represent the distribution of peak intensities for samples collected during the dry and rainy seasons, with individual observations shown as jittered points. White circles indicate estimated marginal means derived from linear mixed-effects models, and black error bars represent 95% confidence intervals back-transformed to the original scale

To further interrogate the multivariate structure underlying these patterns, a PLS-DA was performed using metabolite intensities adjusted for individual-level variation. This analysis revealed a partial and structured separation between seasons (Fig. 2a), indicating that seasonal differences emerge from the combined variation of multiple metabolites rather than a single dominant feature. The validity of this model was confirmed by permutation tests (Fig. S5a).

Metabolites contributing most strongly to group separation were identified based on variable importance in projection (VIP > 1). When considered jointly with the mixed-effects models, this approach highlighted the metabolites P2, P21, P1, P24, P9, P10, P14, and P7, as well as the megastigmane class, as central to seasonal differentiation (Fig. 2b). These metabolites not only contributed to the multivariate separation but also exhibited consistent directional effects across seasons, reinforcing their role in structuring the observed metabolic shifts (Fig. 2c). Although the metabolite P16 did not exceed the VIP, its value was close to the threshold, suggesting a secondary yet consistent contribution to the multivariate structure.

### Effects of fruiting on foliar metabolic profiles

Fruiting was the phenological factor explaining the largest proportion of metabolic variability in the leaves of *M. albicans* (Fruit: Pseudo-F_1_,_36_ = 8.45, R^2^ = 0.1619, p = 0.0002). PERMDISP analysis indicated that mean distances to the multivariate centroid were highly similar between groups (with fruit = 0.01696; without fruit = 0.01790), and the permutation test did not reveal significant heterogeneity of dispersion (Pseudo-F_1_,_38_ = 0.209, p = 0.640).

**Fig. 3:**
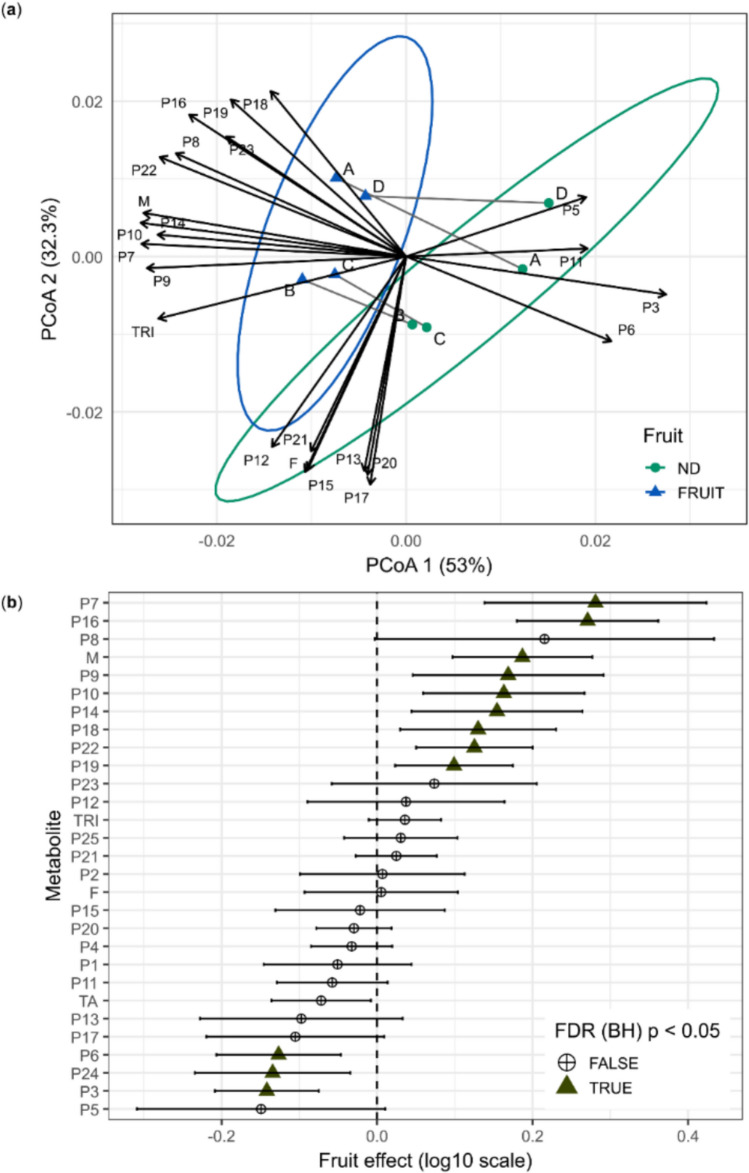
**a** PCoA based on Bray–Curtis distances calculated from metabolite intensities (log_10_ scale), showing the separation between fruiting (FRUIT) and non-fruiting (ND) individuals. Points represent individual plants (A, B, C, or D), connected by grey lines to indicate paired sampling over time. Vectors indicate metabolites correlated with the ordination of axes, with vector length and direction proportional to the strength and direction of the association. **b** Forest plot of the fixed effects of fruiting estimated using linear mixed-effects models (log_10_ scale), with individual included as a random effect. Points represent effect estimates, and horizontal bars indicate 95% confidence intervals based on the t distribution. Positive values indicate higher mean intensities in fruiting individuals, whereas negative values indicate higher mean intensities in non-fruiting individuals. Metabolites showing significant effects after multiple-testing correction using the Benjamini–Hochberg method (FDR p < 0.05) are indicated by triangles; circles denote non-significant effects. The annotations of the compounds (P1-P25) are described in Table 1. *F* flavonoids, *M* megastigmanes, *TA* hydrolysable tannins, *TRI* triterpenes

The structure of multivariate patterns was explored using a PCoA based on Bray–Curtis distances, constructed from the median metabolic intensities per individual and phenological state (fruiting or non-fruiting) (Fig. 3a). The multivariate ordination obtained by PCoA (PCoA1 = 53.0%; PCoA2 = 32.3%) revealed a clear separation between fruiting and non-fruiting individuals, accompanied by low within-group dispersion, indicating high multivariate similarity within each phenological state. This pattern suggests that fruiting is associated not only with changes in the central tendency of metabolic composition but also with a reduction in multivariate variability among individuals, as evidenced by the dispersion structure observed in ordination space.

From the validated mixed-effects models, we extracted the estimated fixed effect of fruiting for each detected metabolite to evaluate the influence of this factor at the univariate level. These effect estimates were summarized in a forest plot with Wald 95% confidence intervals, and p values adjusted using the Benjamini–Hochberg procedure (FDR) (Fig. 3b). Metabolites with significant effects were identified, and their boxplots are shown in Fig. S6.

Metabolite-specific analyses revealed 11 compounds with significant differences according to phenological state (Fig. 3b). Ellagic acid derivatives (P18 and P19), megastigmanes (P9, P10, P14, and P16), *C*-procyanidin NHTP–HHDP–hexoside (P7), and O-hexosyl triterpene (P22) showed positive fruiting effects, corresponding to higher mean intensities in fruiting individuals compared with non-fruiting ones. When metabolite classes were compared, only megastigmanes exhibited a significant effect contrast, showing increased class-level intensity during fruiting. All detected megastigmanes followed a consistent trend. In contrast, in the absence of fruits, metabolites P3 (hexahydroxydiphenoyl nonahydroxytriphenoy-hexoside), P6 (a hydrolysable tannin), and the unidentified peak (P24) displayed higher intensities.

**Fig. 4:**
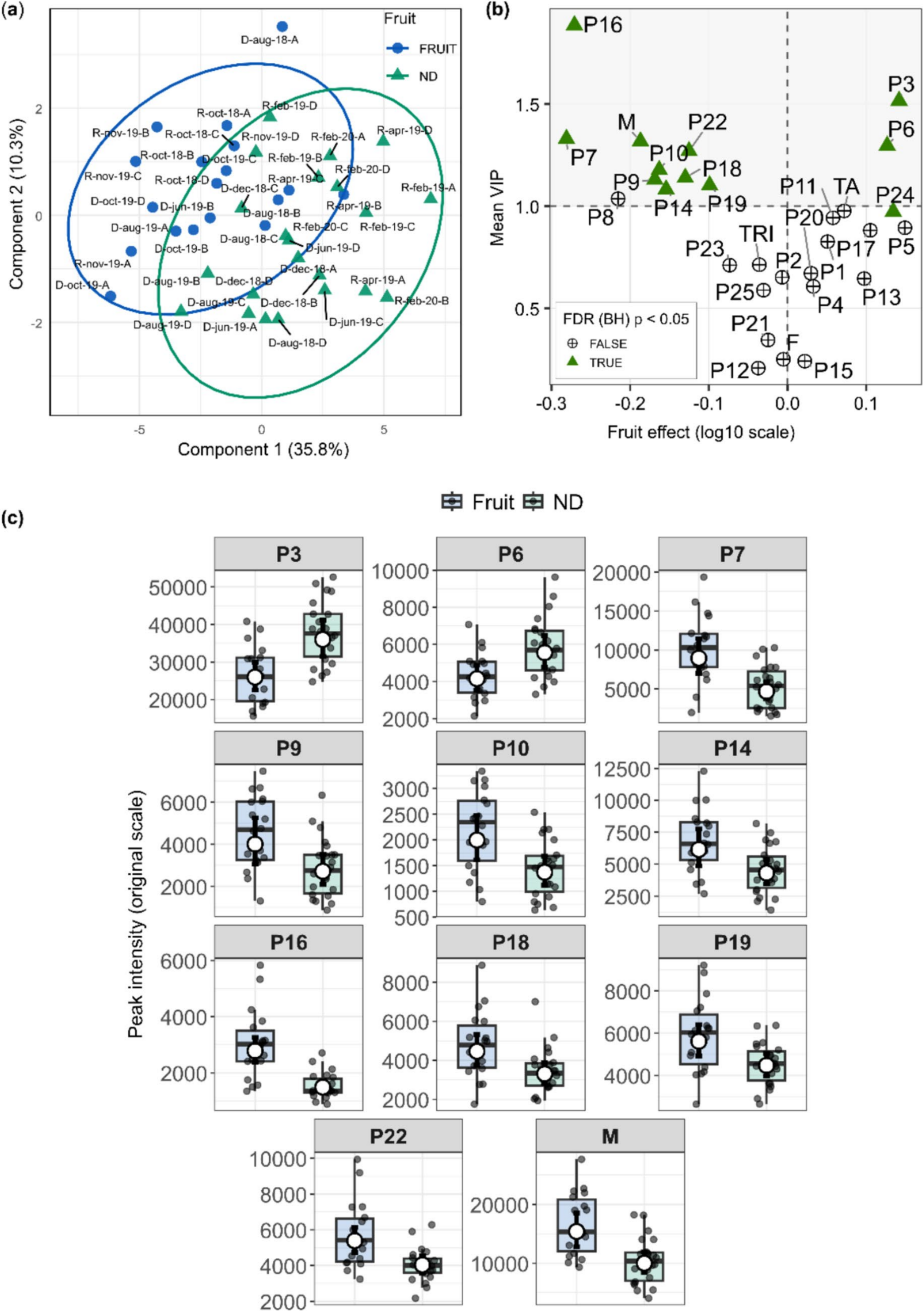
Metabolic variation associated with fruiting in *Miconia albicans*. **a** PLS-DA score plot based on metabolite intensities adjusted for individual-level effects, showing a partial separation between fruiting (blue) and non-fruiting (green) individuals. Ellipses represent 95% confidence regions. The first two components explain the proportion of variance indicated on the axes. **b** Integration of multivariate and univariate results. Mean VIP scores (y-axis) are plotted against the estimated fruiting effect (fruiting–non-fruiting contrast, log10 scale; x-axis) derived from linear mixed-effects models. Metabolites above the horizontal dashed line (VIP > 1) that remained significant after FDR correction (Benjamini–Hochberg, p < 0.05) were considered relevant for multivariate discrimination. Metabolites significant after FDR correction are highlighted with green triangles. **c** Boxplots showing the distribution of peak intensities for fruiting and non-fruiting individuals. Points represent individual observations. White circles indicate estimated marginal means from linear mixed-effects models, and black error bars represent 95% confidence intervals back-transformed to the original scale

To further examine how fruiting structured the metabolic profile at the multivariate level, a PLS-DA was performed using metabolite intensities adjusted for individual-level effects. The ordination revealed a partial but discernible separation between fruiting and non-fruiting individuals (Fig. 4a), indicating that phenological state contributes to the overall organization of leaf metabolism, although with some overlap between groups. The validity of this model was confirmed by permutation tests (Fig. S5b).

Metabolites contributing most strongly to this separation were identified based on VIP scores and then interpreted jointly with the mixed-effects models (Fig. 4b). This combined analysis largely corroborated the univariate results (Fig. 4c), highlighting megastigmanes (P9, P10, P14, and P16), ellagic acid derivatives (P18 and P19), *C*-procyanidin NHTP–HHDP–hexoside (P7), O-hexosyl triterpene (P22), and the megastigmane class (M) as major contributors to the discrimination between fruiting and non-fruiting individuals. In the opposite direction, P3 and P6 were associated with non-fruiting plants, consistent with their negative fruiting effects in the mixed models.

In contrast, the compound P24, despite showing a significant effect in the mixed-effects models, did not exhibit a strong contribution to the multivariate discrimination (VIP < 1). This discrepancy indicates that, although P24 responds to fruiting when evaluated in isolation, its variation is not aligned with the main covariance structure driving group separation.

### Limited metabolic effects associated with budding

**Fig. 5:**
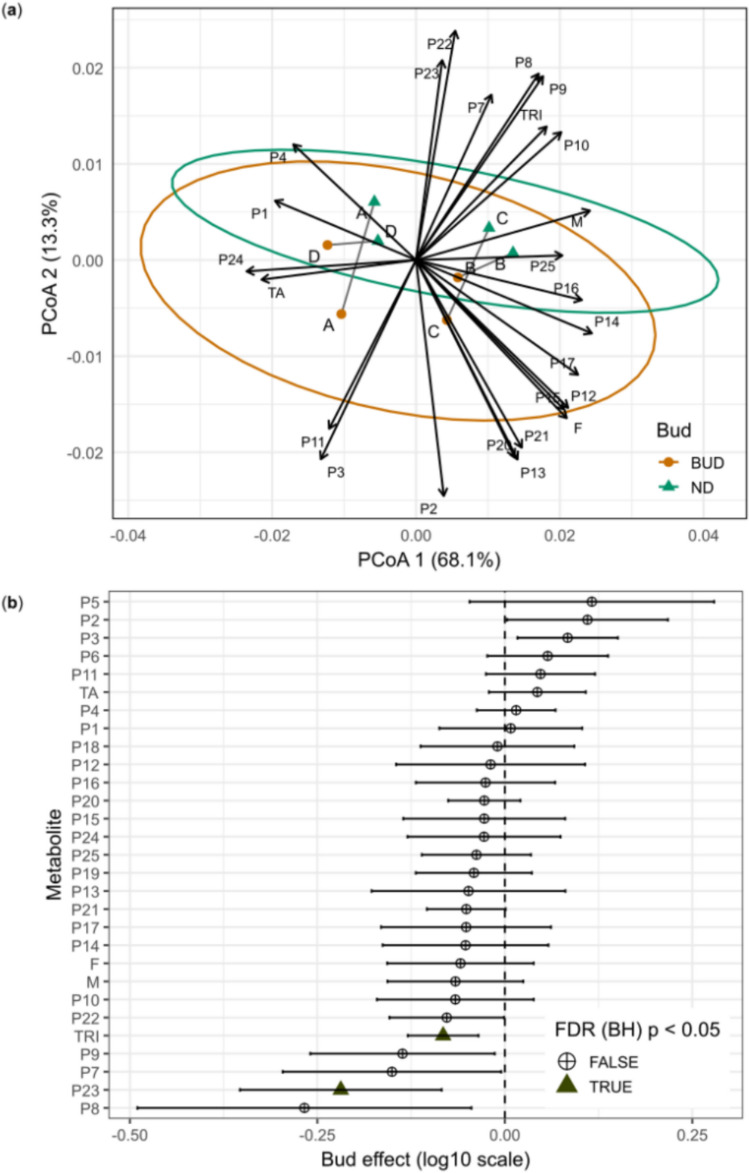
**a** Principal coordinates analysis (PCoA) based on Bray–Curtis distances calculated from metabolite intensities (log_10_ scale), showing the separation between budding (BUD) and non-budding (ND) individuals. Points represent individual plants (A, B, C, or D), connected by grey lines to indicate paired sampling over time. Ellipses represent 95% confidence intervals for each group. Vectors indicate metabolites correlated with the ordination of axes, with vector length and direction proportional to the strength and direction of the association. **b** Forest plot of the fixed effects of budding estimated using linear mixed-effects models (log_10_ scale), with individual included as a random effect. Points represent effect estimates, and horizontal bars indicate 95% confidence intervals based on the t distribution. Positive values indicate higher mean intensities in budding individuals, whereas negative values indicate higher mean intensities in non-budding individuals. Metabolites showing significant effects after multiple-testing correction using the Benjamini–Hochberg method (FDR p < 0.05) are indicated by triangles; circles denote non-significant effects. The annotations of the compounds (P1-P25) are described in Table 1. *F* flavonoids, *M* megastigmanes, *TA* hydrolysable tannins, *TRI* triterpenes

PERMANOVA detected a significant, albeit small, effect of budding on the multivariate metabolic composition of *M. albicans* leaves (Bud: Pseudo-F_1_,_36_ = 2.67, R^2^ = 0.0511, p = 0.0290). PERMDISP analysis did not indicate differences in multivariate dispersion between groups (Pseudo-F_1_,_38_ = 0.010, p = 0.913). The structure of multivariate patterns, explored through a PCoA based on Bray–Curtis distances, did not reveal separation between samples with and without budding, which overlapped in ordination space (Fig. 5a). These results indicate that budding is associated with subtle multivariate shifts, in agreement with the low proportion of total variability explained by this factor.

Linear mixed-effects models were used to evaluate whether budding exerted a fixed effect on the univariate profiles of metabolites detected in the leaves. Estimates of the budding effect were summarized in a forest plot with Wald 95% confidence intervals, and p values adjusted using the Benjamini–Hochberg procedure (FDR) (Fig. 3b). Metabolites with significant effects were identified, and their boxplots are shown in Fig. S6.

Univariate model-based analyses showed that most metabolites had small effect estimates with wide and non-significant confidence intervals. Only the triterpene (arjunolic acid) (P23) showed a significant univariate effect, with higher intensities in the leaves of non-budding individuals. Consistently, the triterpene class was the only one to display a univariate trend associated with budding, suggesting that the effect of this phenological factor can be restricted to this chemical class.

**Fig. 6:**
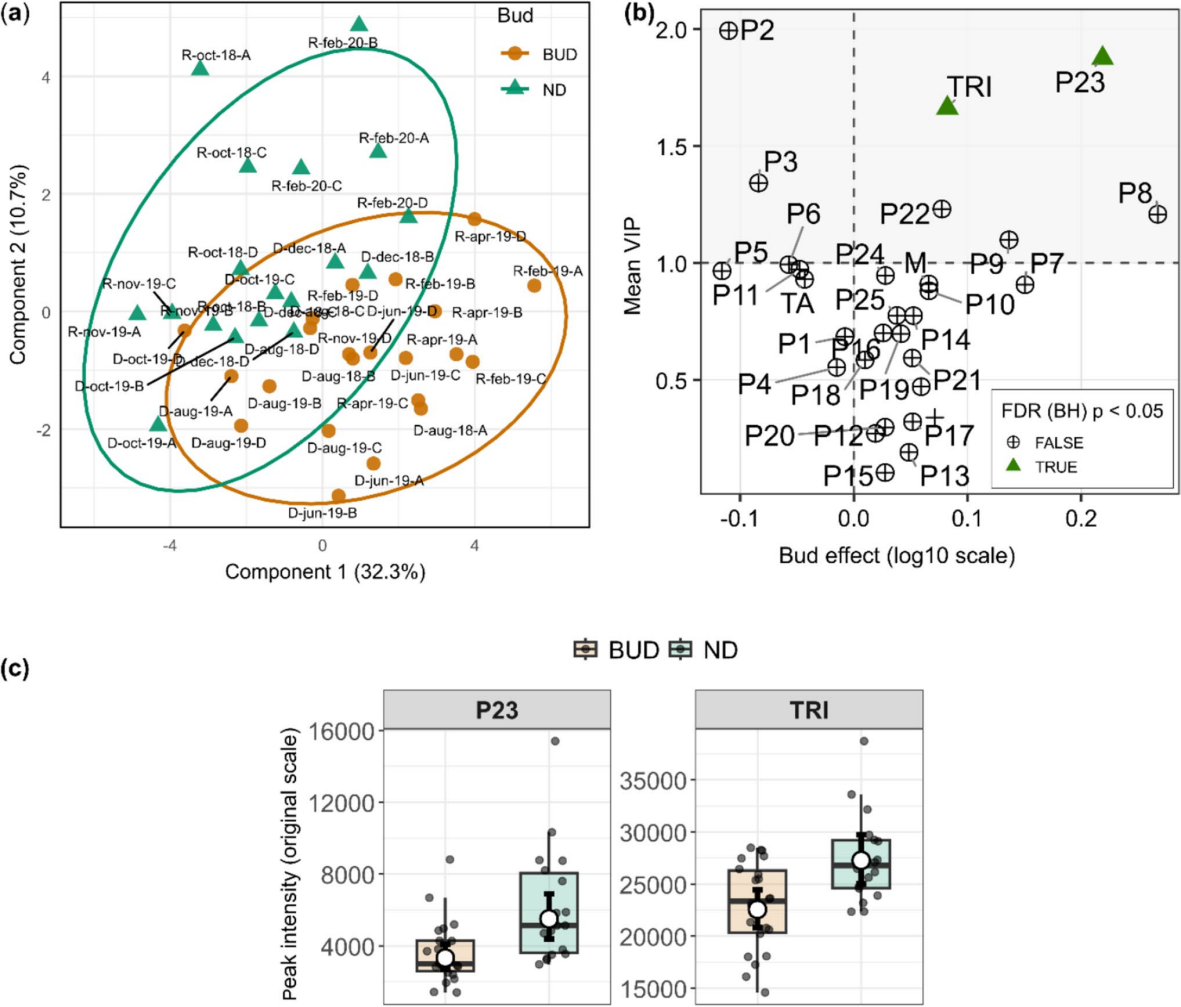
**a** PLS-DA score plot based on metabolite intensities adjusted for individual-level effects, showing a modest separation between budding (orange) and non-budding (green) individuals. Ellipses represent 95% confidence regions. The first two components explain the proportion of variance indicated on the axes. **b** Integration of multivariate and univariate results. Mean VIP scores (y-axis) are plotted against the estimated budding effect (budding–non-budding contrast, log10 scale; x-axis) derived from linear mixed-effects models. Metabolites above the horizontal dashed line (VIP > 1) that remained significant after FDR correction (Benjamini–Hochberg, p < 0.05) were considered relevant for multivariate discrimination. Metabolites significant after FDR correction are highlighted with green triangles. **c** Boxplots showing the distribution of peak intensities for selected metabolites across budding and non-budding individuals. Points represent individual observations. White circles indicate estimated marginal means from linear mixed-effects models, and black error bars represent 95% confidence intervals back-transformed to the original scale

To investigate the multivariate structure associated with budding, a PLS-DA was performed using metabolite intensities adjusted for individual-level effects. The score plot revealed only a modest separation between budding and non-budding individuals (Fig. 6a), indicating that this phenological factor exerts a relatively weak influence on the global metabolic profile. The validity of this model was confirmed by permutation tests (Fig. S5c).

Based on VIP scores (VIP > 1), a larger set of metabolites appeared to contribute to the multivariate discrimination (Fig. 6b). However, when these results were contrasted with the mixed-effects models, only a subset of these features was supported by statistically robust univariate effects. In particular, the triterpene P23 and the triterpene class (TRI) were the only metabolites consistently associated with budding across both analytical approaches (Fig. 6c).

This discrepancy suggests that part of the multivariate signal captured by the PLS-DA reflects covariance among metabolites rather than robust, independent responses to budding. Importantly, it also highlights the susceptibility of PLS-DA to overfitting, particularly when the underlying biological signal is weak or diffuse. In such cases, the model may identify apparent group separation and assign high importance to variables that do not exhibit consistent effects when tested individually. The integration with mixed-effects models therefore acts as a critical validation step.

## Discussion

The foliar specialized metabolism of *M. albicans* is structured by multiple, partially independent sources of variation. Seasonality in the Brazilian savanna (Cerrado), fruiting, and budding exerted significant effects on metabolic composition, although their effect sizes and expression differed between multivariate and univariate analyses.

Seasonality accounted for a moderate proportion of global metabolic variation (9.7%). Independently, PERMDISP revealed significant differences in multivariate dispersion between seasons (p < 0.05), with greater distances to group centroids observed during the rainy season than during the dry season.

The observed differences in the structure of metabolic variability suggest the presence of contrasts in the degree of inter-individual variability associated with seasonality. These patterns have been discussed within the framework of the plasticity–canalization axis of molecular phenotypes, according to which abiotic stress conditions tend to promote more homogeneous metabolic profiles among individuals, whereas less restrictive conditions are associated with increased phenotypic heterogeneity, without necessarily implying the absence of differentiation between environmental states (Alseekh et al., 2025; Shaar‐Moshe et al., 2019; Steward et al., 2023). Although our data do not allow a direct test of this axis, the observed differences in sample-to-centroid distances illustrate shifts in the structure of metabolic heterogeneity that are consistent with this conceptual framework, which has been widely discussed in other systems.

The observed multivariate dispersion in foliar metabolism does not imply the absence of seasonal regulation at the metabolite-specific level. Univariate mixed-effects models revealed that several metabolites exhibited significant seasonal responses. Among the chemical classes detected, megastigmanes (M) were the only class for which all annotated metabolites (P9, P10, P14, and P16) showed higher intensities during the dry season.

Megastigmanes are C_13_ apocarotenoids derived from the oxidative cleavage of carotenoids, generated through both enzymatic pathways mediated by carotenoid cleavage dioxygenases and non-enzymatic processes associated with photo-oxidative conditions (Ramel et al., 2012, 2013). These routes converge on C_13_ apocarotenoid intermediates that are subsequently modified by redox reactions and conjugation processes, including glycosylation and acylation, giving rise to the structural diversity of megastigmanes detected in leaves (El-Sayed et al., 2025). In this study, all detected megastigmanes correspond to highly oxygenated and conjugated derivatives (*O*-hexosides and esterified by gallic acid), including roseoside (P9), trihydroxy megastigmane *O*-hexoside (P10), mallophenol B (P14), and clypearoside A (P16), which show a structural profile characteristic of stabilized end-products of carotenoid oxidative turnover rather than transient volatile intermediates.

Although megastigmanes are not regarded as primary antioxidants of the photosynthetic apparatus, experimental evidence indicates that specific derivatives, such as mallophenol B, exhibit measurable radical-scavenging activity, attributable to their conjugated structural modifications, particularly the presence of galloyl groups (Matsunami et al., 2006). The formation and stabilization of these compounds may indirectly contribute to oxidative stress responses, reflecting both enhanced carotenoid turnover and the integration of redox-signaling processes mediated by carotenoid oxidation products (El-Sayed et al., 2025; Ramel et al., 2012). Accordingly, the enrichment of megastigmanes during the dry season in *M. albicans* is consistent with a scenario of recurrent seasonal stress, although the specific biosynthetic pathways and signaling steps involved cannot be resolved from the present dataset.

Beyond megastigmanes, other metabolites also exhibited individual seasonal responses. Among the compounds showing higher abundance during the dry season, *C*-procyanidin NHTP–HHDP–hexoside tannin (P7) displayed a localized increase. Similarly, a glycosylated triterpene (P21) showed higher intensities under dry-season conditions. The concomitant increase in citric acid (P2) during this period further indicates that seasonality influences not only specialized metabolism but also components of central metabolism, potentially reflecting seasonal reconfigurations of cellular energy balance and redox status (Tahjib-Ul-Arif et al., 2021).

To complement these results, multivariate analysis using PLS-DA indicated that seasonal variation is not driven by a single dominant metabolite but rather by the coordinated contribution of multiple features. The strong agreement between metabolites identified as important in the multivariate space and those showing significant effects in mixed-effects models reinforces the consistency of the seasonal signal, indicating that both covariance structure and metabolite-specific responses converge to describe seasonal metabolic reorganization.

Fruiting was the factor explaining the largest proportion of multivariate metabolic variability in the leaves of *M. albicans* (16.2%). The fruiting effect was characterized by low multivariate dispersion among samples according to fruiting status. This pattern suggests a metabolic convergence associated with the reproductive state, consistent with systemic physiological adjustments triggered by investment in reproduction, even in tissues not directly involved in fruit development.

At the univariate level, fruiting exerted significant effects on multiple metabolites, including ellagic acid derivatives (P18 and P19), megastigmanes (P9, P10, P14, and P16), tannins (P3, P6, and P7), as well as a glycosylated triterpene (P22) and an unknown metabolite (P24). The predominance of positive contrasts in the linear mixed-effects models indicates that the reproductive state is associated with higher mean intensities of specific metabolites in leaves, reflecting a reconfiguration of specialized metabolism during fruiting.

Megastigmanes (M) exhibited a consistent class-level response, with all detected compounds showing higher mean intensities in fruiting individuals. The recurrence of this pattern across different axes of variation indicates that megastigmanes occupy a central position in the foliar metabolic dynamics of *M. albicans*. Fruiting is associated with systemic changes in resource allocation and source-sink relationships, which can propagate to the metabolism of vegetative tissues (Chang & Zhu, 2017). These adjustments may involve changes in redox balance and in pathways related to carotenoid turnover, processes widely recognized as integrators of development and responses to environmental stresses (Ramel et al., 2012). In this context, the enrichment of megastigmanes in the leaves of fruiting individuals is consistent with a systemic activation of apocarotenoid metabolism associated with reproductive development, followed by the stabilization of these compounds through redox modifications and conjugation processes.

From a functional ecological perspective, the coordinated accumulation of megastigmanes across seasonal and reproductive contexts suggests that this class may integrate multiple signals related to stress and development, linking abiotic constraints and life-cycle transitions to foliar chemical phenotypes. This pattern is visually reinforced in the heatmap of model-adjusted marginal means (Fig. S6), which shows higher estimated intensities of megastigmanes under fruiting and dry-season conditions. Such integration is consistent with the role of apocarotenoid metabolism as an interface between redox homeostasis, signaling, and resource allocation (El-Sayed et al., 2025; Ramel et al., 2012, 2013).

Ellagic acid derivatives (P18 and P19) also exhibited a consistent response to reproductive status, with higher mean intensities in fruiting individuals. Considering that ellagic acid and its methylated derivatives are chemically consistent with products derived from the hydrolysis and remodeling of ellagitannins (Grundhöfer et al., 2001; Haslam, 2007), this pattern suggests that the fruiting may be associated with a reconfiguration of hydrolysable tannin metabolism in leaves. Two high-molecular-weight ellagitannins, P3 (nonahydroxytriphenoyl (NHTP)-hexahydroxydiphenoyl(HHDP)–hexoside) and P6 (a hydrolysable tannin), showed lower intensities in fruiting individuals, whereas simpler ellagic acid derivatives (P18 and P19) increased under the same context. This contrast is consistent with the hypothesis that fruiting involves a redistribution of the hydrolysable tannins in leaves, potentially mediated by hydrolysis, depolymerization, or structural remodeling processes, rather than a unidirectional regulation of biosynthesis across the entire class. In parallel, the increase in P7 (*C*-procyanidin nonahydroxytriphenoyl (NHTP)-hexahydroxydiphenoyl(HHDP) tannin) suggests that structurally hybrid compounds, combining condensed and hydrolysable units, may be differentially retained or synthesized during the reproductive state.Budding was the factor with the smallest impact on the multivariate variance of foliar metabolism in *M. albicans*, explaining 5.1% of the variation. Although no significant differences in multivariate heterogeneity were detected, the ordination shows substantial overlap between individuals with and without shoot emergence, suggesting that any metabolic differences associated with this factor are subtle at the global scale. At the univariate level, the triterpene arjunolic acid showed a detectable association with budding, exhibiting lower mean intensities in individuals without evident shoots. The triterpene class displayed a similar pattern in the mixed-effects models. This pattern suggests that budding may be associated with quantitative adjustments restricted to the triterpene pathway, affecting the overall intensity of this metabolic class rather than promoting coordinated changes across the foliar metabolome as a whole.

## Conclusion

The untargeted metabolomic approach revealed that foliar metabolism in *M. albicans* is structured by multiple factors associated with both the pronounced seasonality in the Brazilian savanna (Cerrado) and the phenological state of individuals. Fruiting emerged as the factor with the greatest magnitude, being associated with a global metabolic reorganization and affecting specific chemical classes, notably megastigmanes and tannins. In contrast, seasonality exerted a more selective effect, acting predominantly on the megastigmanes, which exhibited higher intensities during the dry season. Budding, in turn, showed the smallest multivariate effect, with responses restricted to the triterpene pathway and a single metabolite.

The recurrence of megastigmane responses, observed consistently at the class level and confirmed for all metabolites in univariate analyses, establish this class as a central component of foliar metabolic plasticity in *M. albicans*. The systematic increase in megastigmane intensities in both fruiting individuals and during the dry season indicates that this class responds convergently to distinct axes of environmental and phenological variation, reflecting integrated metabolic adjustments associated with life-cycle transitions and recurrent seasonal conditions. To our knowledge, this study provides the first evidence of the systematic involvement of this metabolic class in foliar chemical dynamics within the genus *Miconia*, as well as of its relevance to the life history of a species from the Brazilian Cerrado.

Taken together, our results consolidate foliar specialized metabolism as a sensitive interface linking environmental conditions, phenological states, and ecological strategies in *M. albicans*. By revealing consistent and hierarchically structured patterns of metabolic variation, this study establishes an empirical basis for integrated interpretations of foliar chemical plasticity. Building on these advances, future investigations integrating controlled experimental approaches, metabolic tracing, and functional data will help to further elucidate the biosynthetic, regulatory, and physiological mechanisms underlying the patterns identified here, particularly with respect to the role of megastigmanes. Moreover, the observed metabolic shifts may be reflected in the pharmacological properties of *M. albicans*, especially during the fruiting period, when tannins and megastigmanes show increased abundances. These metabolite classes have been widely associated with anti-inflammatory activity in literature, suggesting a potential link between phenological state, metabolic composition, and biological efficacy.

## Supplementary Information

Below is the link to the electronic supplementary material.

Supplementary file1 (PDF 1502 kb)

## Data Availability

The raw and processed metabolomics data generated in this study, along with the SIRIUS workspace and compound annotations, have been deposited in the MassIVE repository under the identifier MSV000100861. The dataset will be made publicly available upon publication of the article. During the peer-review process, the data can be accessed using the access key GSTb3YLNh73vw30u in https://massive.ucsd.edu/ProteoSAFe/dataset.jsp?task=8ec12b8578444bbc8cfb14f5f9901969. The GNPS job (molecular networking and library matching results) is publicly available at: https://gnps.ucsd.edu/ProteoSAFe/status.jsp?task=ee35e6a6f209405fa05f4d8491a53058.
