# Supplementary material for: Seasonality and fruiting converge on megastigmanes to shape foliar metabolic plasticity in Miconia albicans (Swartz) Triana (Melastomataceae)
Source: Metabolomics. 2026 May 6;22(3):65. doi: 10.1007/s11306-026-02436-2 (PMC13149598; doi:10.1007/s11306-026-02436-2)
Supplement: Supplementary file 1 — Supplementary file1 (PDF 1502 kb) [file 11306_2026_2436_MOESM1_ESM.pdf]

SUPPLEMENTARY INFORMATION

Seasonality and fruiting converge on megastigmanes to shape foliar metabolic plasticity in *Miconia albicans* (Swartz) Triana (Melastomataceae)

André Nunes Silva[1§], Djaceli Sampaio de Oliveira Dembogurski[1§], Amanda Galdi Boaretto[1], Carlos Alexandre Carollo[1], Flavio Macedo Alves[2], Denise Brentan Silva[1*]

[1]Laboratory of Natural Products and Mass Spectrometry (Lanes), Faculty of Pharmaceutical Sciences, Food and Nutrition (FACFAN), Federal University of Mato Grosso do Sul, 79070-900, Campo Grande-Mato Grosso do Sul, Brazil.

[2]Laboratory of Botany, Biosciences Institute (INBIO), Federal University of Mato Grosso do Sul, 79070-900, Campo Grande-Mato Grosso do Sul, Brazil.

[§]Author with same contribution

ORCID:

A. N. Silva: 0000-0001-5810-1005

D. S. O. Dembogurski: 0000-0002-7422-7238

A. G. Boaretto: 0000-0003-3786-899X

C.A. Carollo: 0000-0003-1231-9441

F. M. Alves: 0000-0001-5634-8266

D. B. Silva: 0000-0003-0872-2756

* Corresponding author: denise.brentan@ufms.br (D.B. Silva).

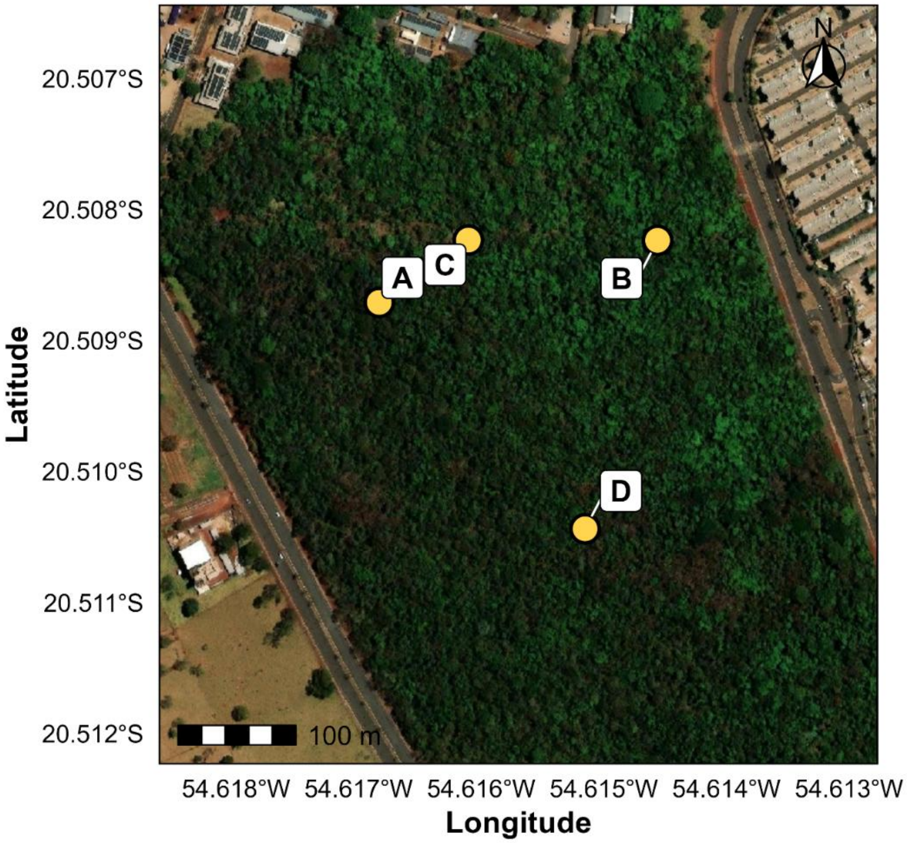

Fig. **S1** Map of sampling locations in the Cerradinho Reserve, Federal University of Mato Grosso do Sul (UFMS), Campo Grande, Brazil. Points A–D correspond to the four georeferenced sampling sites where foliar material of *Miconia albicans* was collected.

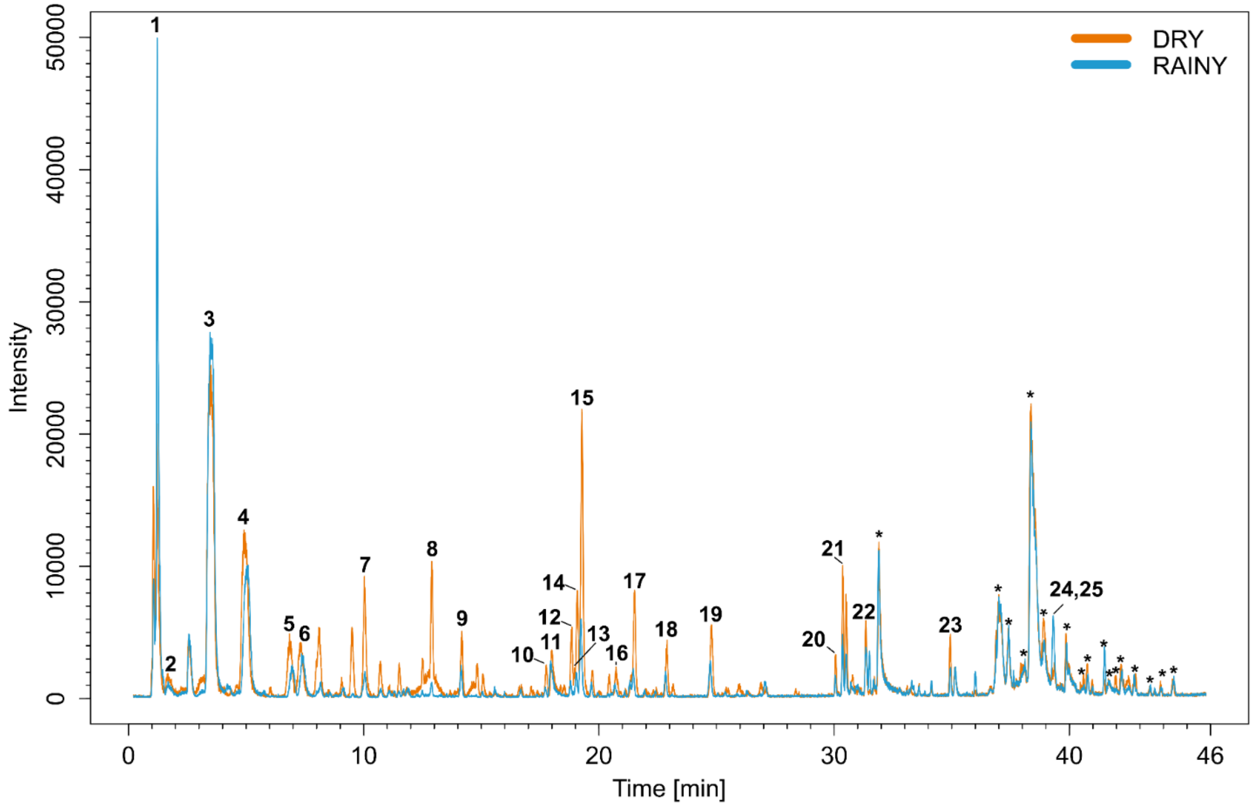

Fig. **S2** Representative base peak chromatograms (BPCs) acquired in full-scan MS and negative ion mode from *Miconia albicans* leaves. The blue trace corresponds to a sample collected during the rainy season, whereas the orange trace represents a sample collected during the dry season. *: peaks detected in blank samples, representing background signals associated with the chromatographic system, solvent impurities, or instrumental sources.

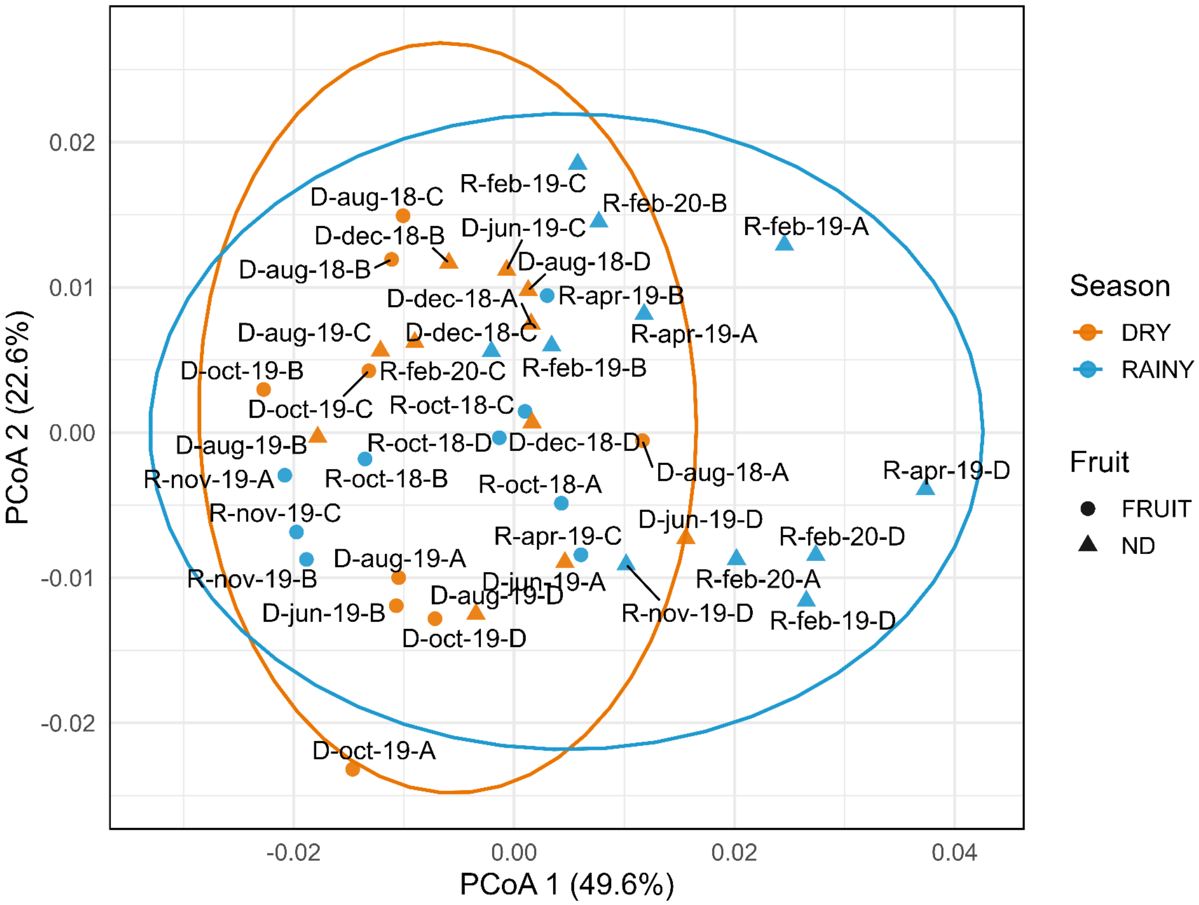

Fig. **S3** Principal Coordinates Analysis (PCoA) based on Bray–Curtis distances calculated from metabolite intensities ($\log_{10}$ scale), showing the separation between samples collected during the dry (DRY) and rainy (RAINY) seasons. Point shapes represent phenological state (circles = fruiting; triangles = non-fruiting, ND).

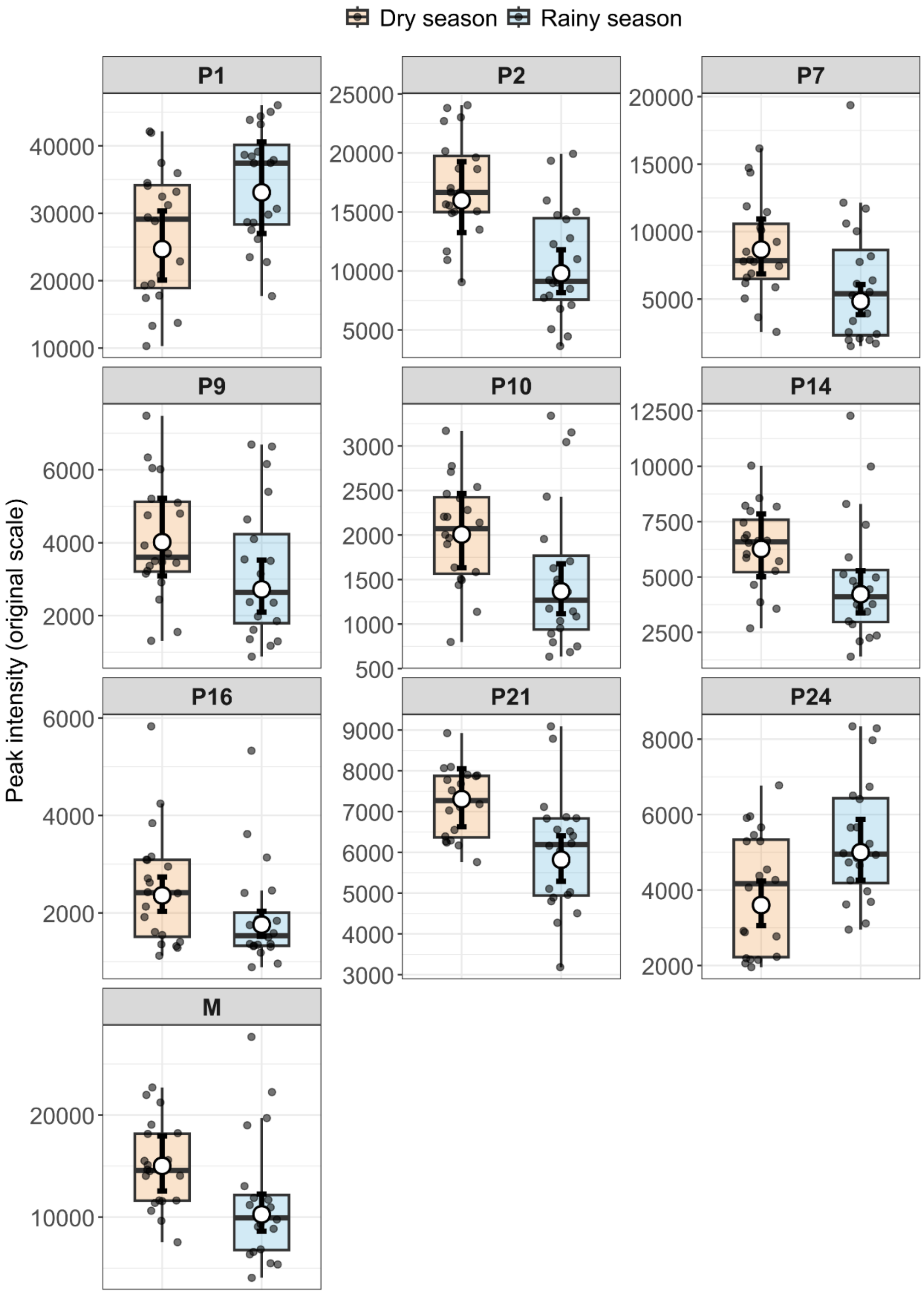

Fig. S4 Effect of season on metabolite intensities. All metabolites presented showed a significant effect of dry and rainy seasons after multiple-testing correction using the Benjamini–Hochberg method (FDR $p < 0.05$) . Boxplots represent the distribution of peak intensities (original scale) for samples collected during the dry and rainy seasons, with individual observations shown as jittered points. White circles indicate estimated marginal means derived from linear mixed-effects models, and black error bars represent 95% confidence intervals back-transformed to the original scale. Models included individuals as random intercept to account for repeated sampling. Facets display each metabolite separately with independent y-axis scaling.

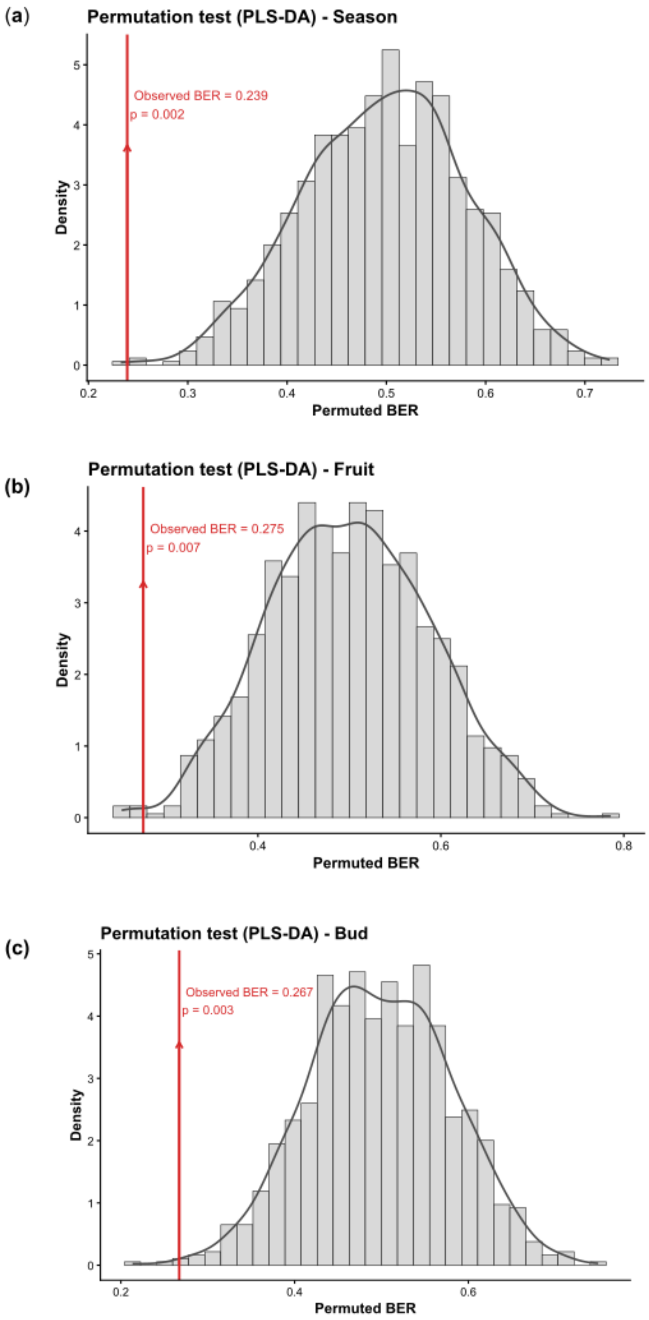

Fig. S5 Permutation tests supporting the statistical significance of PLS-DA models. Permutation tests (n = 999) were used to evaluate the significance of PLS-DA models for (a) Season, (b) Fruit, and (c) Bud. Histograms represent the empirical null distributions of the balanced error rate (BER) obtained from models fitted to randomly permuted class labels, with model performance re-estimated by repeated M-fold cross-validation for each permutation. The vertical red line indicates the observed BER from the original (non-permuted) model. In all cases, the observed BER was substantially lower than the null distribution, indicating that model performance exceeded the expected by chance. Empirical p-values were calculated based on the proportion of permuted models achieving equal or better performance (lower BER) than the observed model.

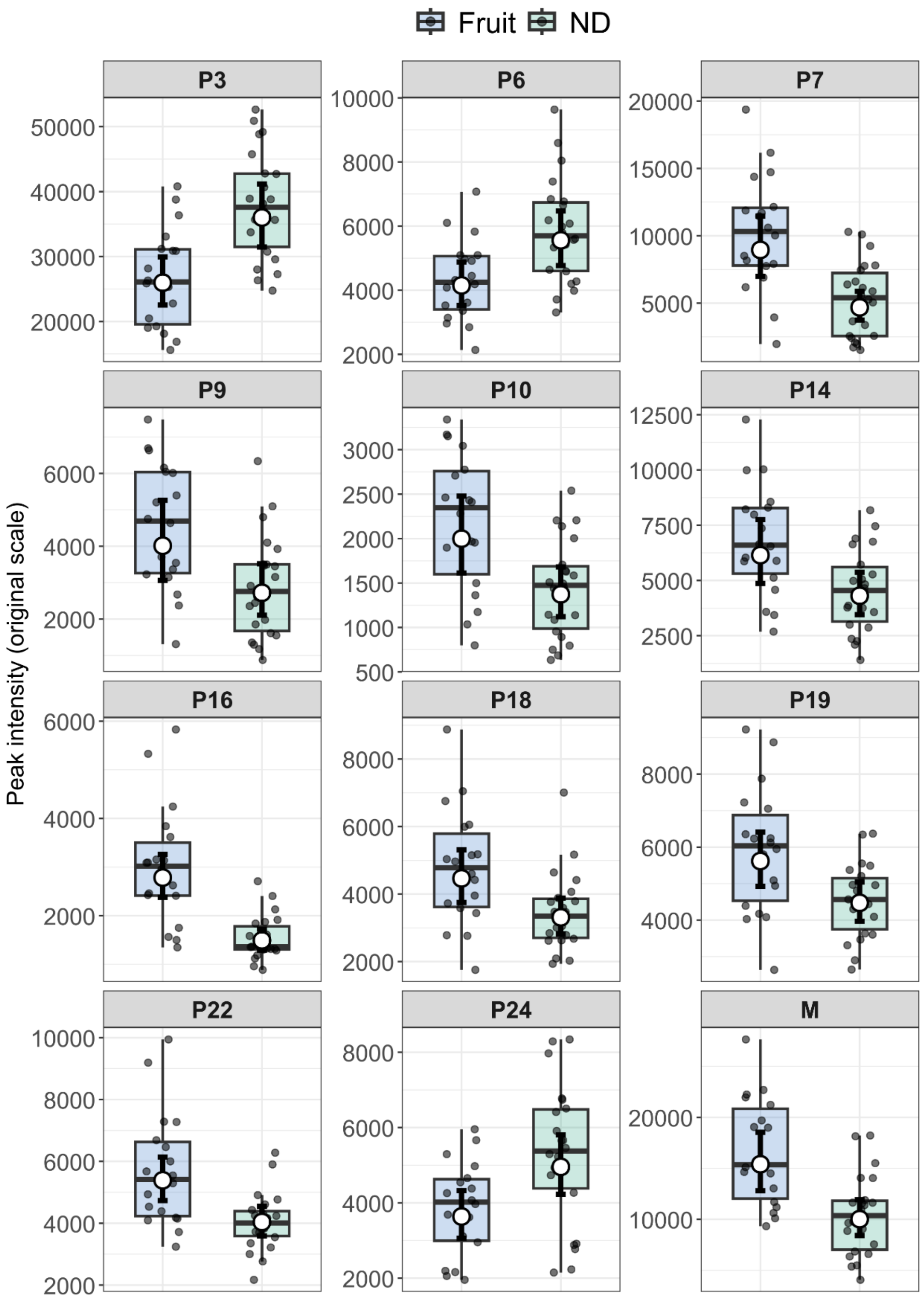

Fig. S6 Effect of fruiting on metabolite intensities. All metabolites presented showed a significant effect of fruiting after multiple-testing correction using the Benjamini–Hochberg method (FDR $p < 0.05$). Boxplots represent the distribution of peak intensities (original scale) for fruiting (FRUIT) and non-fruiting (ND) samples, with individual observations shown as jittered points. White circles indicate estimated marginal means derived from linear mixed-effects models and black error bars represent 95% confidence intervals back-transformed to the original scale. Models included individuals as random intercept to account for repeated sampling over time. Facets display each metabolite separately with independent y-axis scaling.

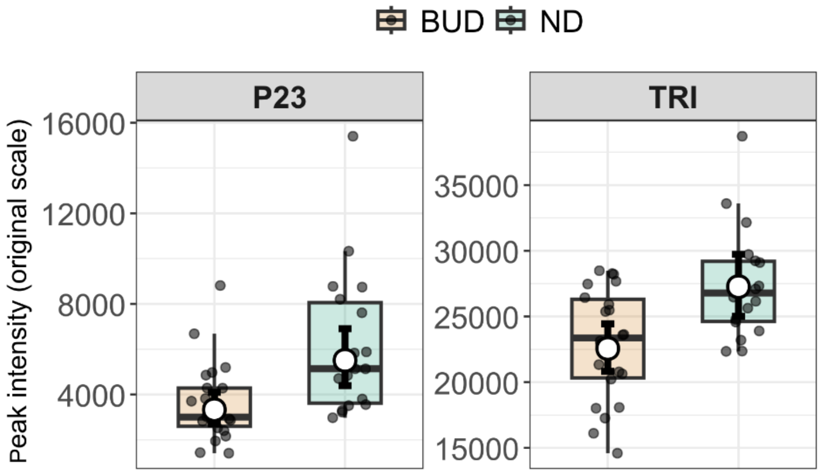

Fig. S7 Effect of budding on metabolite intensities. All metabolites presented showed a significant effect of budding ($p < 0.05$). Boxplots show the distribution of peak intensities (original scale) for budding (BUD) and non-budding (ND) samples, with individual observations displayed as jittered points. White circles represent estimated marginal means obtained from linear mixed-effects models, and black error bars indicate 95% confidence intervals back-transformed to the original scale. Individual was included as a random intercept to account for repeated sampling over time. Each metabolite is presented in a separate facet with independent y-axis scaling.

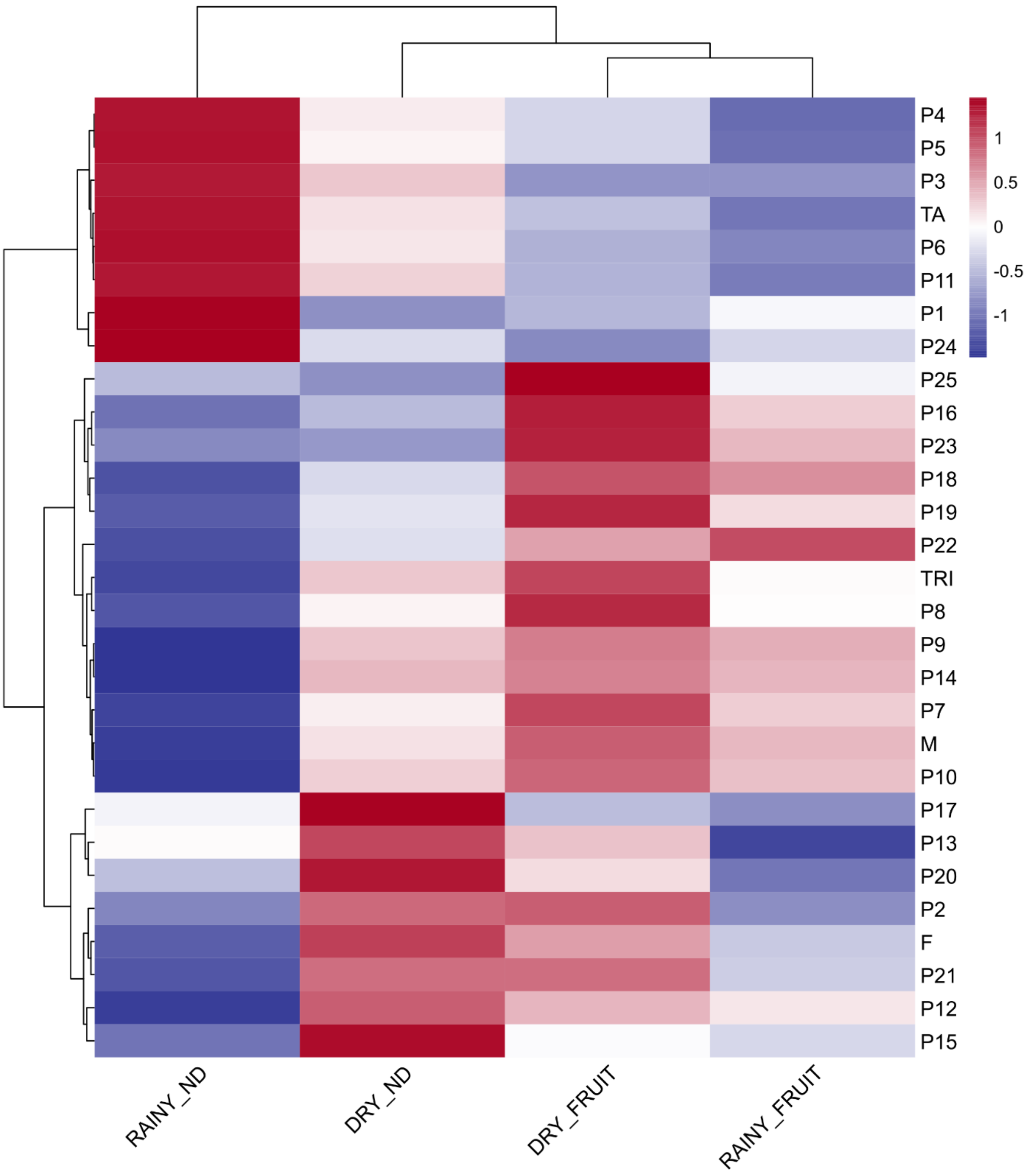

Fig. S8 Heatmap of adjusted metabolite intensities estimated from linear mixed-effects models. For each metabolite, $\log_{10}$-transformed intensities were modeled as a function of the interaction between Season (dry and rainy) and Fruit status (fruiting and non-fruiting), including Bud as a fixed effect and individuals as a random intercept. The heatmap displays the estimated marginal means (emmeans) for each Season × Fruit combination. Values were standardized by metabolite (row-wise z-score) to highlight relative differences among conditions. Rows (metabolites) and columns (Season × Fruit conditions) were hierarchically clustered using Euclidean distance and Ward's method (ward.D2). Color gradients represent relative metabolite abundance, from lower (blue) to higher (red) values.

Table S1. The phenological stages of *M. albicans* individuals collected.

Samples	Season	Fruit	Bud	Flower
R-Oct-18-A	RAINY	FRUIT	ND	ND
R-Oct-18-B	RAINY	FRUIT	ND	ND
R-Oct-18-C	RAINY	FRUIT	ND	ND
R-Oct-18-D	RAINY	FRUIT	ND	ND
D-Oct-19-A	DRY	FRUIT	ND	ND
D-Oct-19-B	DRY	FRUIT	ND	FLOWER
D-Oct-19-C	DRY	FRUIT	ND	ND
D-Oct-19-D	DRY	FRUIT	BUD	FLOWER
R-Nov-19-A	RAINY	FRUIT	ND	ND
R-Nov-19-B	RAINY	FRUIT	ND	ND
R-Nov-19-C	RAINY	FRUIT	ND	ND
R-Apr-19-B	RAINY	FRUIT	BUD	ND
R-Apr-19-C	RAINY	FRUIT	BUD	ND
D-Jun-19-B	DRY	FRUIT	BUD	ND
D-Aug-18-A	DRY	FRUIT	BUD	FLOWER
D-Aug-1A8-B	DRY	FRUIT	BUD	FLOWER
D-Aug-18-C	DRY	FRUIT	BUD	FLOWER
D-Aug-19-A	DRY	FRUIT	BUD	ND
R-Nov-19-D	RAINY	ND	BUD	ND
D-Dec-18-A	DRY	ND	ND	ND
D-Dec-18-B	DRY	ND	ND	ND
D-Dec-18-C	DRY	ND	ND	ND
D-Dec-18-D	DRY	ND	ND	ND
R-Feb-19-A	RAINY	ND	BUD	ND
R-Feb-19-B	RAINY	ND	BUD	ND
R-Feb-19-C	RAINY	ND	BUD	ND
R-Feb-19-D	RAINY	ND	BUD	ND
R-Feb-20-A	RAINY	ND	ND	ND
R-Feb-20-B	RAINY	ND	ND	ND
R-Feb-20-C	RAINY	ND	ND	ND
R-Feb-20-D	RAINY	ND	ND	ND
R-Apr-19-A	RAINY	ND	BUD	ND
R-Apr-19-D	RAINY	ND	BUD	ND

D-Jun-19-A	DRY	ND	BUD	ND
D-Jun-19-C	DRY	ND	BUD	ND
D-Jun-19-D	DRY	ND	BUD	ND
D-Aug-18-D	DRY	ND	BUD	FLOWER
D-Aug-19-B	DRY	ND	BUD	ND
D-Aug-19-C	DRY	ND	BUD	ND
D-Aug-19-D	DRY	ND	BUD	ND

The table summarizes the sampling scheme, indicating for each sample the season of collection (RAINY or DRY) and the phenological status of the individual at the time of sampling, including fruiting (FRUIT or ND), budding (BUD or ND), and flowering (FLOWER or ND). Sample identifiers encode the season (R = rainy; D = dry), month and year of collection, and individual plant (A–D), allowing traceability of repeated, paired sampling across seasons and phenological stages. ND: non-detected

Table S2. Climatic data of the months of sample collection (August 2018 to February 2020).

**Month**	**Season**	**IT**	**MAXT**	**MAINT**	**RA**	**IH**	**MAXH**	**MINH**	**WG**	**DWR**
aug-18	dry	20.9	34.4	7.2	112.2	56.2	96	10	37.7	22
oct-18	rainy	25.9	34.0	17.5	167.4	70.8	97	32	40.9	13
dec-18	dry	25.9	36.0	15.3	55.0	65.9	97	23	35.1	20
feb-19	rainy	25.5	35.8	17.0	271.8	74.9	98	26	33.9	15
apr-19	rainy	24.5	32.6	16.6	104.4	75.5	98	39	29.4	21
jun-19	dry	22.7	31.5	14.0	20.6	57.1	98	19	35.1	27
aug-19	dry	23.2	35.7	6.6	2.0	47.4	92	14	44.0	28
oct-19	dry	26.7	39.6	15.4	30.8	54.9	95	17	42.2	21
nov-19	rainy	26.6	37.6	18.6	149.6	62.7	97	21	38.1	18
feb-20	rainy	25.1	34.2	17.0	227.2	77.6	100	29	32.6	11

Climatic data were obtained from the online database of Mato Grosso do Sul Weather and Climate Monitoring Center. IT: Instantaneous temperature; MAXT: Maximum Temperature; MAINT: Minimum Temperature; RA: Rainfall; IH: Instantaneous Humidity; MAXH: Maximum Humidity; MINH: Minimum Humidity; WG: Wind Gust; DWR: Days Without Rain.

Table S3. Validation of metrics and effect sizes of linear mixed-effects models fitted to metabolites of *M. albicans*.

Metabolite	LRT_p_value	R^2_marginal	R^2_conditional	DHARMa_uniformity_p	DHARMa_dispersion_p	DHARMa_outliers_p	Singular_fit
F	0.1430	0.0668	0.5583	0.3287	0.5440	1.000	0
TA	0.0353	0.1954	0.2281	0.8632	0.8040	1.000	0
TRI	0.0005	0.3677	0.3744	0.9939	0.8360	1.000	0
M	0.0000	0.4985	0.5297	0.9404	0.7800	1.000	0
P1	0.0271	0.1918	0.2809	0.6973	0.7620	1.000	0
P2	0.0001	0.4155	0.4202	0.7288	0.8440	1.000	0
P3	0.0000	0.4587	0.5042	0.2314	0.7780	1.000	0
P4	0.4600	0.0631	0.1214	0.8092	0.7800	1.000	0
P5	0.0657	0.1683	0.1683	0.8299	0.8560	1.000	1
P6	0.0030	0.2983	0.3390	0.6441	0.7880	1.000	0
P7	0.0000	0.5000	0.5000	0.8479	0.8560	1.000	1
P8	0.0030	0.2994	0.2994	0.9981	0.8560	1.000	1
P9	0.0003	0.3673	0.4310	0.8015	0.7700	0.077	0
P10	0.0002	0.3898	0.4311	0.9681	0.7820	0.077	0
P11	0.1239	0.1371	0.1371	0.4793	0.8560	1.000	1
P12	0.2365	0.0686	0.4107	0.2080	0.5900	1.000	0
P13	0.1064	0.0839	0.5227	0.9843	0.5560	1.000	0
P14	0.0005	0.3538	0.4040	0.9979	0.7800	1.000	0
P15	0.3786	0.0393	0.5256	0.0576	0.5460	1.000	0
P16	0.0000	0.5747	0.5747	0.7095	0.8560	1.000	1
P17	0.1361	0.0661	0.5890	0.3613	0.5400	1.000	0
P18	0.0327	0.2007	0.2007	0.9750	0.8560	1.000	1
P19	0.0073	0.2643	0.2643	0.9949	0.8560	1.000	1
P20	0.0720	0.1320	0.3437	0.9750	0.6560	1.000	0
P21	0.0008	0.3427	0.3638	0.9818	0.8060	1.000	0
P22	0.0006	0.3560	0.3560	0.3781	0.8560	1.000	1
P23	0.0039	0.2897	0.2897	0.2777	0.8560	1.000	1
P24	0.0015	0.3255	0.3255	0.7818	0.8560	1.000	1
P25	0.5147	0.0507	0.2197	0.5492	0.7020	1.000	0

Likelihood ratio test p-values (LRT p-value) were used to assess the significance of the fixed effect of interest in each metabolite-specific model. Marginal (R^2_marginal) and conditional (R^2_conditional) R^2 values represent variance explained by fixed effects alone and by the full model (fixed plus random effects), respectively. Model assumptions were evaluated using DHARMa residual diagnostics, including tests for residual uniformity, dispersion, and outliers. The Singular_fit column indicates models in which the random-effects variance approached zero, suggesting boundary (singular) fits. The annotation of the compounds (P1-P25) is described in Table 1 of the manuscript.
